# Canada's Forests Are Shifting From a Recovery‐Driven Carbon Sink to a Disturbance‐Driven Carbon Source

**DOI:** 10.1111/gcb.70958

**Published:** 2026-06-05

**Authors:** Salvatore R. Curasi, Joe R. Melton, Elyn R. Humphreys, Vivek K. Arora, Jason Beaver, Alex J. Cannon, Jing M. Chen, Txomin Hermosilla, Sung‐Ching Lee, Michael A. Wulder

**Affiliations:** ^1^ Canadian Centre for Climate Modelling and Analysis Environment and Climate Change Canada Victoria British Columbia Canada; ^2^ Department of Geography & Environmental Studies Carleton University Ottawa Ontario Canada; ^3^ National Wildlife Research Centre Environment and Climate Change Ottawa Ontario Canada; ^4^ Climate Modeling Division Environment, and Climate Change Canada Victoria British Columbia Canada; ^5^ Department of Geography University of Toronto Toronto Ontario Canada; ^6^ Canadian Forest Service (Pacific Forestry Centre), Natural Resources Canada Victoria British Columbia Canada; ^7^ Department of Biogeochemical Integration Max Planck Institute for Biogeochemistry Jena Germany

**Keywords:** Canada, carbon cycle, carbon sink, CLASSIC, disturbance, land surface model, wildfire

## Abstract

Canada's terrestrial ecosystems are critical to the global carbon cycle and are responding to unprecedented climate change and wildfire disturbance. However, our understanding of Canada's historical (~1920—present) carbon cycle is incomplete. There are also no published physically coherent (i.e., those that respect conservation laws) wall‐to‐wall estimates of all major carbon pools and fluxes for Canada. Existing assessments vary in spatial scale and methodology, yielding notable differences in the magnitude of Canada's land carbon sink. Moreover, inversions and data‐driven estimates do not disentangle the relative influence of disturbance, CO_2_ fertilization, or climate change on Canada's carbon cycle. Here, we synthesize information from the site to Canada‐wide scale with a land surface model and the most comprehensive wildfire and wood harvest estimates available to provide the first physically coherent wall‐to‐wall estimates of all major carbon pools and fluxes for Canada. Using factorial model runs, we show that Canada's terrestrial ecosystems have been a carbon sink since the mid‐20th‐century, due to wildfire and timber harvest before 1940. Since the early 2000s, wildfire disturbance has been driving Canadian forests towards becoming a carbon source. Continued increases in wildfire activity will further weaken, and may ultimately reverse, Canada's role as a carbon sink.

## Graphical abstract

Canada's terrestrial ecosystems have historically functioned as a net carbon sink but are increasingly impacted by wildfire and timber harvest. Using wall‐to‐wall, physically coherent estimates of all major carbon pools and fluxes from the Canadian Land Surface Scheme Including Biogeochemical Cycles land surface model, we show that rising wildfire activity is threatening Canada's land carbon sink.

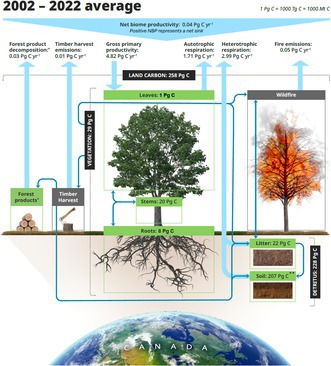

## 1 Introduction

Canada represents around 7% of the world's land mass and contains ecosystems critical to the global carbon (C) cycle, including 31% of boreal forests globally, and 36% of tundra (Curasi, Melton, Arora, et al. 2024; Curasi, Melton, Humphreys, et al. 2024; Jain et al. 2024; Keenan and Williams 2018; Lenton et al. 2008). Canada's ecosystems are responding to unprecedented climate change, increasing atmospheric CO_2_, intensifying wildfires, and anthropogenic activity like wood harvest (Jain et al. 2024; Kurz et al. 2008; Lenton et al. 2008; White et al. 2017). For example, the 2023 wildfire season burned 12.74 Mha, eight times the long‐term average (Pelletier et al. 2024), releasing record C emissions (~700 to 2400 Tg CO_2_) (Byrne et al. 2024; Kirchmeier‐Young et al. 2024). Even low 2023 emissions estimates exceed Canada's 2020–2023 average anthropogenic emissions (~690 Tg CO_2_ e year^−1^) (Byrne et al. 2024; Kirchmeier‐Young et al. 2024; ECCC 2023). The 2023 wildfire season was driven by extreme weather (Jain et al. 2024; Lai and Zhang 2025) due to climate change (Kirchmeier‐Young et al. 2024). Our understanding of wildfire and disturbance impacts on Canada's terrestrial C budget is limited. There are no published physically coherent (i.e., those that respect conservation laws) wall‐to‐wall (i.e., encompassing the entire pan‐Canadian domain, including forested and unforested land) estimates of all major C pools and fluxes (Smyth, Metsaranta, Tompalski, et al. 2024; Smyth, Metsaranta, Fortin, et al. 2024). A better understanding of Canada's terrestrial C cycle will inform Canada's Climate Science 2050 framework priorities (ECCC 2024; UNFCCC 2015), and quantify how changes in CO_2_ uptake by Canada's natural ecosystems impact net‐zero GHG emissions goals. The historical responses of these ecosystems also foreshadow how Canada's C cycle will respond to future climate change.

Vegetation takes up atmospheric CO_2_ through photosynthesis (Chapin III et al. 2006). Some of this C is quickly respired to meet metabolic demand, while the remainder is stored in biomass. When vegetation sheds biomass or dies, this C becomes litter and then humified soil organic C. Litter and soil C are in part respired back to the atmosphere by soil biota. However, C accumulates and persists in soils for thousands of years (Sanborn 2016). Disturbances such as wildfire and wood harvest have both immediate and long‐term impacts that both release stored C and enhance C uptake (Chapin III et al. 2006; Goulden et al. 2011; Schulze et al. 1999). Wildfire and harvest immediately release vegetation and soil C to the atmosphere. Fire and harvest release labile C to litter and soil as incompletely burned necromass and logging residues, which is respired over longer timescales (González‐Pérez et al. 2004). Wood harvest sequesters C in wood products, where residence time depends upon the product's lifetime. Atmospheric CO_2_ uptake gradually returns as vegetation communities recover after disturbance (Amiro et al. 2010). The timescales and magnitude of disturbance effects create complex successional source‐sink dynamics.

Gaining a comprehensive understanding of Canada's terrestrial C cycle is challenging because existing assessments may not be physically coherent, differ in their spatial scales, temporal coverage, and methodological approaches (Table S1) (Chen, Chen, Liu, Cihlar, and Gray 2000; Chen, Chen, Liu, and Cihlar 2000; Chen et al. 2003; Girardin et al. 2016; Gonsamo et al. 2013; Kurz et al. 2008; Marchand et al. 2018; Smyth, Metsaranta, Tompalski, et al. 2024; Smyth, Metsaranta, Fortin, et al. 2024; Zhao et al. 2021). They focus on only select state variables (Sothe et al. 2022) or portions of the landscape (e.g., managed (Kurz et al. 2008) or forested (Chen et al. 2003)). Relatively coarse resolution process‐based models and inversion point to a net C sink in Canada over the last two decades (Poulter et al. 2025). Notably, there are large differences in the magnitude of Canada's C sink between top‐down (i.e., atmospheric inversions) and bottom‐up (i.e., physical modeling‐based) approaches (Deng et al. 2022; Keenan and Williams 2018; O'Sullivan et al. 2024; Zhao et al. 2021).

Approaches, such as inventory‐based estimates and data‐driven models, can simulate the Canadian C cycle at spatial resolutions of kilometers or less (Gonsamo et al. 2013; Jung et al. 2020; Sothe et al. 2022). However, data‐driven approaches require large amounts of high‐quality training data, which may not capture all C fluxes and pools, especially at large scales. Atmospheric inversions provide a robust top‐down perspective on net C fluxes at global scales but become less robust at regional scales (Peylin et al. 2013). Moreover, inversions, data‐driven, and inventory‐based estimates may struggle to separate different processes' contributions to total atmosphere‐land CO_2_ exchange and to make future projections (Deng et al. 2022; Kurz et al. 1995, 2008; Kurz and Apps 1999; Shiga et al. 2018; Xiao et al. 2014).

A comprehensive synthesis of Canada's historical terrestrial C cycle using process‐based modeling is needed to fill these knowledge gaps. Unlike other approaches, process‐based models explicitly represent key processes and their response to novel climate conditions. They can independently reconstruct trends in the terrestrial C cycle, provide physically consistent wall‐to‐wall estimates of major C pools and fluxes, and disentangle different processes' impacts using factorial analysis (Arora et al. 2023, 2013; Curasi, Melton, Arora, et al. 2024; Curasi, Melton, Humphreys, et al. 2024; Jones et al. 2016; Kou‐Giesbrecht and Arora 2022). These capabilities have rarely been used over Canada as land surface models (LSMs) operate at coarse spatial resolution (0.5–1 degree) without Canada‐specific vegetation and disturbance drivers (Friedlingstein et al. 2019; Hayes et al. 2012; Huntzinger et al. 2012; Seiler et al. 2022; Yue et al. 2018). Recently, models tailored to Canada have been developed (Curasi et al. 2023; Curasi, Melton, Arora, et al. 2024; Curasi, Melton, Humphreys, et al. 2024). Here, we present a process‐oriented synthesis of Canada's historical terrestrial C cycle. We estimate pan‐Canadian C fluxes and pools and elucidate the processes controlling Canada's net C balance to understand the system's future responses.

We use the Canadian Land Surface Scheme Including Biogeochemical Cycles (CLASSIC), an LSM tailored to Canada (Curasi, Melton, Arora, et al. 2024; Curasi, Melton, Humphreys, et al. 2024). CLASSIC runs at the site, Canada‐wide and global scale. It's used in the Global Carbon Projects assessment framework (Friedlingstein et al. 2022; Sitch et al. 2024) and is the land surface component of the Canadian Earth System Model (Swart et al. 2019). Unlike the global version (Seiler et al. 2021, 2022), the Canada domain uses Canada‐specific geophysical fields and plant functional types and represents boreal disturbance and high‐latitude processes at 0.22° spatial resolution (Curasi, Melton, Arora, et al. 2024; Curasi, Melton, Humphreys, et al. 2024; Kirchmeier‐Young et al. 2024; Meyer et al. 2021). CLASSIC captures subgrid‐scale heterogeneity, stand recovery, and stand‐replacing wildfire and wood harvest disturbance using dynamic tiling, but not nonstand‐replacing disturbance (i.e., insects, windthrow, selective logging practices). CLASSIC simulates C fluxes across forested and nonforested, managed and unmanaged land (Chen, Chen, Liu, Cihlar, and Gray 2000; Chen, Chen, Liu, and Cihlar 2000; Chen et al. 2003; Kurz et al. 1995, 2008). Unlike preexisting approaches (Chen, Chen, Liu, Cihlar, and Gray 2000; Chen, Chen, Liu, and Cihlar 2000; Chen et al. 2003; Deng et al. 2022; Girardin et al. 2016; Gonsamo et al. 2013; Jung et al. 2020), it outputs coherent spatially explicit wall‐to‐wall estimates of all major C pools and fluxes (15+ fields) at annual to subhourly timesteps. We evaluate CLASSIC's simulated response to fire and wood harvest using a novel site‐level approach. Then we model Canada's net C balance using an ensemble of disturbance trajectories reconstructed from the most comprehensive historical data available (Beaver et al. 2025). We analyze the model's sensitivity to different forcings through factorial analysis and evaluate the model against independent reference data, building a systematic understanding of Canada's historical terrestrial C budget. Finally, we synthesize pan‐Canadian estimates of the natural C pools and fluxes within Canada.

## 2 Methods

### 2.1 Classic

CLASSIC is an open‐source community model and the successor to the coupled Canadian Land Surface Scheme and Canadian Terrestrial Ecosystem Model (CLASS‐CTEM) (Melton et al. 2020). CLASSIC has been extensively evaluated at the site and global scale (Melton et al. 2020; Seiler et al. 2021). It has also been evaluated through contributions to external intercomparisons, including the Fire Model Inter Comparison Project (Burton et al. 2023) and the Global Carbon Project (Friedlingstein et al. 2022). CLASSIC has undergone continuous development work, updates, and improvements since v1.0 (Asaadi et al. 2018; MacKay et al. 2022; Meyer et al. 2021).

The CLASSIC configuration used herein is derived from extensive work to tailor and evaluate the model in the Canada domain (Curasi et al. 2023; Curasi, Melton, Arora, et al. 2024; Curasi, Melton, Humphreys, et al. 2024; Wang et al. 2023). In this configuration, CLASSIC runs on a 0.22‐degree grid, uses 14 biogeochemical plant functional types tailored to represent mid to high‐latitude Canadian genera (Curasi et al. 2023), and uses landcover fractions derived from region‐specific data (Wang et al. 2023). The land cover fractions of the different PFTs are prescribed Canada‐wide (i.e., do not shift dynamically in response to climate during the simulation) in line with our focus on evaluating a segment of the past, and the disturbance forcings used herein (Beaver et al. 2025). The model uses a multilayer soil C model which incorporates a parameterization for respiration and turbation (cryo and bio), optimized using data assimilation (Gauthier et al. 2024).

It uses wildfire and wood harvest disturbance forcings (i.e., per‐grid cell fractions of harvested and burned area read in from a file derived from remotely‐sensed observations) (Curasi, Melton, Arora, et al. 2024; Curasi, Melton, Humphreys, et al. 2024; Kirchmeier‐Young et al. 2024) to drive its representation of disturbance. It represents subgrid‐scale heterogeneity resulting from disturbance using dynamic tiles configured with 12 dynamic tiles with tile optimization as configured and evaluated by Curasi, Melton, Arora, et al. (2024); Curasi, Melton, Humphreys, et al. (2024). The wildfire model parameterization used herein accounts for fire emissions from boreal soils and litter (Curasi, Melton, Arora, et al. 2024; Curasi, Melton, Humphreys, et al. 2024; Kirchmeier‐Young et al. 2024). However, the Canada domain CLASSIC model does not explicitly represent peatland C cycle processes, their associated soil C stocks, and their role in boreal fire emissions from soil.

The dynamic tiling scheme is adapted to facilitate comparison to inversions within the Canadian domain. Dynamic tiling (i.e., splitting and combining subgrid tiles to represent disturbance and succession) occurs at the annual time step as in Curasi, Melton, Arora, et al. (2024); Curasi, Melton, Humphreys, et al. (2024). However, once a subgrid tile is designated for burning, fire occurs at the daily time step. The annual burned area is disaggregated based upon an average monthly climatology of total burned area Canada‐wide from the Global Fire Emissions Database version 4.1, with small fires (2001–2015) (Giglio et al. 2013) and the European Space Agency Climate Change Initiative FireCCI50 MODIS‐derived burned area (2001–2019) (Chuvieco et al. 2018), and the length of each month. This approach facilitates comparisons of monthly NBP between inversions and the model, even though satellite‐derived and historical burned area drivers for Canada are only available annually (Beaver et al. 2025; Hermosilla et al. 2015).

Based upon our site‐level simulations, we built upon the parametrization of Curasi et al. (2023); Curasi, Melton, Arora, et al. (2024); Curasi, Melton, Humphreys, et al. (2024). We proportionally increased the fractions of green leaf biomass released as CO_2_ during fire (woody = 0.67 nonwoody = 0.82, unitless), the fraction of green leaf biomass converted to litter during fire (woody = 0.32, nonwoody = 0.17, unitless), the fraction of brown leaf mass released as CO_2_ during fire (0.9, unitless), the fraction of brown leaf mass converted to litter during fire (0.09, unitless), the fraction of stems biomass released as CO_2_ (Needleleaf trees and shrubs = 0.165, broadleaf PFTS = 0.125, unitless), and the fraction of stem biomass converted to litter (Needleleaf trees and shrubs = 0.83, broadleaf PFTS = 0.87, unitless). The changes improve model consistency with the ecology of the sites and are intended to explicitly represent disturbance events as stand‐replacing and implicitly represent standing dead trees resulting from disturbance (Goulden et al. 2006). We increased the maximum rate of carboxylation, V_cmax_, by 11% to align with the multisite mean from Qu et al. (2023), and utilized a new value of gamma within the range of observational uncertainty (Arora et al. 2009) (0.5, unitless). Gamma is a unitless parameter that empirically represents the down‐regulation of GPP increases with CO_2_ fertilization because of progressive nutrient limitation (Arora et al. 2009). This value of Gamma is consistent with the degree of down‐regulation due to progressive nutrient limitation observed experimentally in seedlings though limited experimental and observational data is available for Canadian genera (18% in Bigras and Bertrand (2006); see also table 2 in Arora et al. (2009)). Across the range of 1900–2023 atmospheric CO2 concentrations (289–420 ppm) which are significantly more constrained than high CO_2_ high warming future scenarios (1135 ppm under SSP585) (Meinshausen et al. 2020) the full range of down regulation parameters in Arora et al. (2009) would alter the modeled effect by just ~8%. Finally, we reduced the basal, maintenance, and growth respiration rates for all pools by 25% in line with the site‐level fluxes. The CLASSIC source code and Canada domain parameterization used herein are archived on Zenodo (see code and data availability below).

### 2.2 Site‐Level Forcings and Observational Data

We evaluate CLASSIC at 26 forested flux tower sites across Canada (Pastorello et al. 2020). The sites have between 2 and 20 years of available meteorological data and eddy flux observations. The flux tower data are primarily from FLUXNET2015, the efforts of Fluxnet Canada, and the Canadian Carbon Program (FLUXNET Canada Team 2016; Pastorello et al. 2020) (Table S2). Prior comparisons between CLASSIC and flux tower data, especially for NEP are confounded by the lack of long‐term meteorological observations to spin up the model to equilibrium and represent the historical period (taken as 1700 to the start of observations) and the fact that a handful of sites may be influenced by recovery from disturbances (e.g., CZ‐BK1 and CG‐Tch in Melton et al. (2020)). The latter is particularly significant for sites within Canada, many of which are chrono sequences (Goulden et al. 2006, 2011; Humphreys et al. 2006; Mkhabela et al. 2009; Peichl and Arain 2006). We address these issues by extending the meteorological time series using bias‐corrected reanalysis and explicitly representing disturbance (i.e., fire or harvest).

CLASSIC requires seven meteorological forcing variables: incoming shortwave radiation, incoming longwave radiation, air temperature, precipitation rate, air pressure, specific humidity, and wind speed. Suitable 30‐min gap‐filled meteorological drivers are available from the flux towers during the observation period (Pastorello et al. 2020). For the spin‐up and historical period (1700—the start of tower observations), we use bias‐corrected, interpolated, and disaggregated reanalysis created via the methods described by Meyer et al. (2021).

The 1901–1978 portion of the forcing comes from the Inter‐Sectoral Impact Model Intercomparison Project GSWP3–W5E5, and the 1979–2018 portion comes from the ERA5 time series bias corrected to match the means of the overlapping period in the GSWP3–W5E5 (ECMWF 2019; Kim 2017; Lange 2019, 2020a, 2020b). The values are taken from the grid cell nearest the site and then bias‐corrected to match the tower observations using an N‐dimensional probability function transform (Cannon 2018). This methodology matches the marginal distributions and multivariate dependence structure of the in situ observations while preserving the trends in reanalysis. The resulting daily values are then disaggregated to a 30‐min time step following the methods of Meyer et al. (2021) and Melton and Arora (2016). Before 1901 (1700–1901), the forcing loops the first 25 years from the Inter‐Sectoral Impact Model Intercomparison Project GSWP3–W5E5. The model is also driven by 1700–2023 atmospheric CO_2_ concentrations from the Global Carbon Project (Friedlingstein et al. 2022).

We prescribe the fractional coverage of PFTs based on each site's published field survey data (Table S2). The plant functional types within CLASSIC represent the broad patterns of vegetation across the Canadian landscape (Curasi et al. 2023), but not more granular species or population‐level differences (Curasi et al. 2019; Qu et al. 2023), which could influence the correspondence between predicted and observed fluxes. Information about fire and harvest years was obtained from each site's related publications (Table S2). For sites where disturbance occurred far in the past or is not precisely known, we infer the year of disturbance from published stand age estimates (Beaver et al. 2025; Chen, Chen, Liu, Cihlar, and Gray 2000; Chen, Chen, Liu, and Cihlar 2000; Kurz and Apps 1999).

### 2.3 Site‐Level Simulation Evaluation

We carry out two simulations, one with disturbance (factual) and one without disturbance (counterfactual) per site to isolate and illustrate the impact of disturbance at the site level and evaluate the model. Neither of these simulations uses dynamic tiling, as this feature does not apply to simulations of stand‐replacing disturbance events at the site level. For both simulations, we spin up the model to equilibrium conditions corresponding to the year 1700 and then do a transient run from 1700 to the start of each site's flux tower observations. During spin‐up, we loop the earliest 25 years of bias‐corrected meteorology available (1901–1925) and hold atmospheric CO_2_ concentrations constant at the preindustrial (1700) level. The 1700–1900 portion of the transient run uses looping of the 1901–1925 climate, but transient atmospheric CO_2_ concentrations. After 1900, both transient meteorology and atmospheric CO_2_ were used. In the factual simulation, fire and harvest are simulated during the transient run to occur in the corresponding year from the site's disturbance history, whereas in the counterfactual simulation, fire and harvest are ignored (i.e., no disturbance occurs). Finally, the model is run using the observed tower meteorology and compared to the flux tower observations.

We directly compare the predictions and observations (Figure S1) and evaluate the model against a selection of variables available from the flux tower, including aboveground biomass (AGB), gross primary productivity (GPP), ecosystem respiration (ER), and net ecosystem productivity (NEP). To isolate the simulated impact of disturbance and better understand its legacy on the landscape, we calculate a site‐level response metric via the change in the mean absolute error (ΔMAE; Equation 1) using the predicted (*x*_predicted_) and tower observed (*x*_observed_) values from the factual and counterfactual runs.

$$∆{\mathrm{MAE}}_{i,j}={\mathrm{MAE}}_{i,j,\text{counterfactual}}-{\mathrm{MAE}}_{i,j,\text{factual}};$$

(1) $${\mathrm{MAE}}_{i,j,k}=\frac{\sum_{l=1}^{n}\left|x_{i,j,k,l,\text{predicted}}-x_{i,j,k,l,\text{observed}}\right|}{n};$$

*i* = site ID; *j* = GPP, ER, NEP, AGB. *k* = counterfactual, factual; *n* = number of observations.

This response metric isolates the effect of representing disturbance on the correspondence between the model and data. Given that we are employing a space‐for‐time substitution approach (Likens 2012), this response metric weights the sites equally regardless of the length of the observational record.

### 2.4 Canada Domain Model Forcings and Gridded Reference Data Sets

We obtained a meteorological forcing based on the Climate Research Unit and Japanese reanalysis (CRU‐JRA), interpolated to the CLASSIC model grid. The Global Carbon Project provides this forcing for its TRENDY model intercomparison (Friedlingstein et al. 2022), and its application to CLASSIC is described in detail by Wang et al. (2023). This data set has recently been extended into the year 2023.

We prescribe disturbance across the historical period using fire and harvest drivers developed by Beaver et al. (2025), which are an improvement upon the drivers used by Curasi, Melton, Arora, et al. (2024); Curasi, Melton, Humphreys, et al. (2024). In brief, these drivers integrate multiple permutations of a wide array of data sets detailing stand age (Maltman et al. 2023), fire, and harvest based on satellite observations, vector records, and aspatial records (Hermosilla et al. 2016; Skakun et al. 2021; Van Wagner 1988). They also integrate assumed trajectories of disturbance before reliable historical information was available (Beaver et al. 2025; Chen, Chen, Liu, Cihlar, and Gray 2000; Chen, Chen, Liu, and Cihlar 2000). The algorithm integrates these data sets into a cohesive product by infilling known disturbance events based upon spatial records in a manner that is consistent with remotely sensed stand age estimates (Beaver et al. 2025). These drivers consist of four scenarios representing possible permutations of relevant observational data and assumptions about the historical period. These scenarios differ in particular before 1918, when neither spatial nor aspatial Canada‐wide disturbance data are available. Therefore, these scenarios encompass a range of competing assumptions, including average disturbance levels (“mean” scenarios in Figure S5) and elevated estimates based on stand age (“inferred” scenarios in Figure S5), allowing us to quantify the model's sensitivity to these assumptions. Tree ring or lake charcoal paleo reconstructions, for example, may eventually allow for further evaluation of the true pre‐1918 wildfire trends within this range (Remy et al. 2018). Two scenarios are based on remotely sensed fire and harvest observations from 1985 to 2020, whereas the other relies on vector data sources, which indicate slightly higher disturbance levels across Canada (0.7 Mha higher annually on average from 1985 to 2020). From 2023, remotely sensed harvest is not yet available, so harvest was omitted in that year. Vector burned area records were sourced from the National Burned Area Composite time series (Skakun et al. 2022) and the National Burned Area Composite M3 interim product (Jain et al. 2024). Raster burned area records were sourced from the National Terrestrial Ecosystem Monitoring System (Pelletier et al. 2024).

Two of the scenarios repeat 1920 to 1930 mean Canada‐wide burned area from 1918 to 1745, whereas the other two use disturbance inferred from 1920s stand age (3.2 Mha higher annually on average from 1900 to 1920). In our model runs, we treat each scenario as equally likely and evaluate the impact of the resultant assumptions on the model output. In the early years, when observations are unavailable, the model results agree with the broad historical patterns of fire Canada‐wide, simulated by an independent modeling approach driven by climate, population density, and climatological lightning (Figure S4e) (Curasi, Melton, Arora, et al. 2024).

The fractional coverage of 14 PFTs is derived from Wang et al. (2023), which was expanded upon by Curasi et al. (2023). It integrates information from the North American Land Change Monitoring System land cover (Latifovic et al. 2017), the National Terrestrial Ecosystem Monitoring System (Hermosilla et al. 2016, 2018), satellite‐derived maps of the National Forest Inventory attributes (Beaudoin et al. 2018), and British Columbia's biogeoclimatic ecosystem classification map (MacKenzie and Meidinger 2018) and is referenced to the year 2010. The resulting PFTs represent all terrestrial ecosystems in Canada, including agricultural land, tundra, and peatlands, in addition to forests using appropriate PFTs, including C3 and C4 crops, grasses, sedges, and shrubs. The boundary conditions for each grid cell include soil texture and permeable depth provided by SoilGrids250m (Hengl et al. 2017).

We evaluate our historical runs against independent raster reference data sets. These detail four variables relevant to biogeochemical cycles (Table S1) and include soil C, above‐ground biomass, fire emissions, and net biome productivity. The processing of these data sets is detailed by Seiler et al. (2021, 2022) and Curasi et al. (2022); Curasi, Melton, Arora, et al. (2024); Curasi, Melton, Humphreys, et al. (2024).

### 2.5 Canada Domain Simulations and Evaluations

As with the site‐level runs for each combination of forcings, we first spin up the model to equilibrium using conditions corresponding to the year 1750, then conduct a transient run from 1750 to 2017. During spinup, we loop the first 25 years of the available meteorological driver and hold atmospheric CO_2_ concentrations at the 1750 value. During the transient runs, before 1900, we continue to loop the first 25 years of meteorological data, but use time‐varying atmospheric CO_2_ concentrations and apply fire and harvest with dynamic tiling. During the 1900 to 2023 portion of the transient run, we use time‐varying climate, atmospheric CO_2_ concentrations, fire, and harvest with dynamic tiling. We run four simulations with all possible disturbance drivers. We also conduct fourteen additional factorial simulations to understand the impact of different drivers on the model outputs, following the methods of Kou‐Giesbrecht and Arora (2022) (Appendix S2). All plotting and analyses were carried out in R (Hijmans et al. 2015; R core team 2013). We tested the Canada‐wide and forest net biome productivity time series for significant negative trends using 15‐year regression windows from 1920 to 2023.

## 3 Results

### 3.1 Fire and Harvest Have Large Impacts on Site‐Level Fluxes Canada‐Wide

**FIGURE 1:**
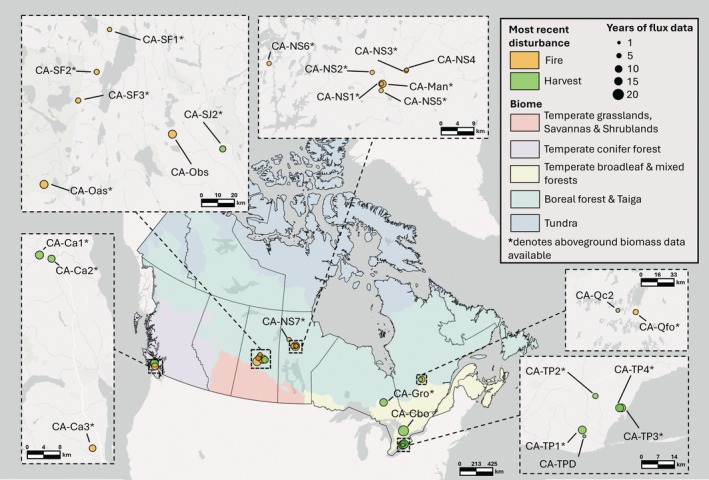
Map of flux tower sites across Canada used in site‐level evaluations. The Biome background map is sourced from Dinerstein et al. (2017).

**FIGURE 2:**
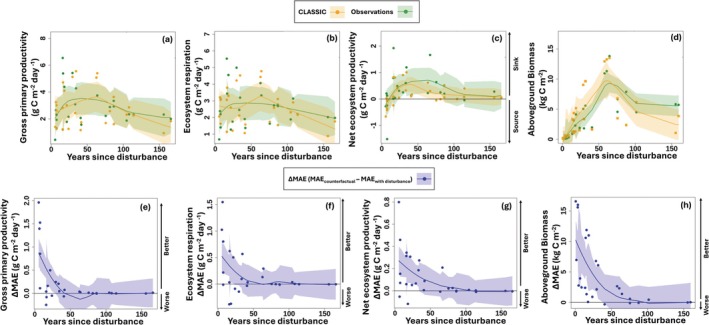
Modelled and observed fluxes and biomass for a chronosequence of sites across Canada. (a) gross primary productivity, (b) ecosystem respiration, and (c) net ecosystem productivity from eddy flux towers (number of sites = 26), as well as (d) above‐ground biomass from site‐level inventories (number of sites = 21). The observations are plotted against the number of years since disturbance (wildfire or harvest) at the site alongside simulations from the Canadian Land Surface Scheme Including Biogeochemical Cycles, which explicitly represent disturbance. Plots of the difference in mean absolute error (ΔMAE) between counterfactual simulations (i.e., simulations without disturbance) and simulations with disturbance for (e) gross primary productivity, (f) ecosystem respiration, and (g) net ecosystem productivity from eddy flux towers (number of sites = 26) as well as (h) above‐ground biomass from site level inventories (number of sites = 21). Positive ΔMAE indicates improved model performance when including disturbance. Each point represents the average for an individual site summarised using loess smoothed regression lines with shaded 95% confidence intervals.

CLASSIC accurately represents C fluxes and the effects of fire and harvest at 26 forested flux tower sites (Figure 1; Table S2). We do simulations that represent disturbance (factual) using site‐observed meteorology and geophysical fields and compare them to the observations. Space‐for‐time substitution uses sites at different stages of development to examine long‐term ecological processes (Likens 2012). We treat these sites, which broadly represent Canada's forest (Figure 1), as a space‐for‐time substitution. Gross primary productivity (GPP), ecosystem respiration (ER), and aboveground biomass (AGB) increase for ~50 years after disturbance, and then plateau (Figure 2a,b,d). Net ecosystem productivity (NEP) transitions from a C source after disturbance to a sink, which weakens and plateaus (Figure 2c). After disturbance, CLASSIC simulates 95% confidence intervals that overlap observations for GPP, ER, AGB, and NEP (Figure 2a–d). CLASSIC realistically simulates disturbance recovery and is well correlated with observed daily fluxes and aboveground biomass (Figure S1; Pearson *r* from 0.47–0.80).

We also used simulations without disturbance (counterfactual) to isolate the impacts of wildfire and wood harvest at the site level. The change in mean absolute error (ΔMAE; Figure 2e–h; Equation 1) between factual (Figure 2a–d) and counterfactual simulations (Figure S2) captures the profound impact of disturbance on ecosystem recovery dynamics and the timescales of those impacts. Positive ΔMAE denotes greater agreement between the model and observations when disturbance is included, whereas negative values denote degradation. For GPP, ER, NEP, and AGB, ΔMAE peaks at recently disturbed sites and approaches zero after 50 years (Figure 2e–h). This shows CLASSIC explicitly captures the well‐understood transition of forests from a C source following disturbance and subsequent recovery present in the observations (Figures 2 and S2) (Goulden et al. 2006, 2011; Mkhabela et al. 2009; Peichl and Arain 2006). The predominance of positive ΔMAE indicates that representing disturbance generally yields greater agreement between simulated results and observations (Figure 2e–h). These results underscore the need to represent disturbance effects on NEP when validating process‐based models against observations or statistically upscaling tower data. Extrapolating from flux tower observations to large scales without accounting for disturbance could introduce biases (Jung et al. 2011, 2020; Keenan and Williams 2018).

In the context of space‐for‐time substitution, we must consider that the sites inhabit a wide climate space and several forest types (Figure 1; Table S2). Thus, the observed temporal patterns (Figure 2a–d) may be influenced by nontemporal factors (Damgaard 2019; Likens 2012; Lovell et al. 2023) like species composition. For example, CLASSIC underestimates AGB at the oldest sites (Figure 2d). However, CLASSIC reproduces Canada‐wide AGB well compared to remotely sensed estimates (Figure S3). Moreover, these sites encompass evergreen and deciduous forests in coastal and boreal Canada, but not permafrost‐affected regions, peatlands, and alpine terrain. The ΔMAE results (Figure 2e–h) control for nontemporal factors by isolating the effect of representing disturbance using factual and counterfactual runs. The modelled and observed patterns are, ecologically, consistent with a broad‐scale signal of disturbance in Canadian forests (Goulden et al. 2006, 2011; Mkhabela et al. 2009; Peichl and Arain 2006). CLASSIC uses plant functional types (PFTs) to represent broad groupings of species (Curasi et al. 2023; Melton et al. 2020; Qu et al. 2023). This evaluation is consistent with the selection of PFTs in CLASSIC and how they balance realism and parsimony (Curasi et al. 2023; Melton et al. 2020; Qu et al. 2023). Collectively, these space‐for‐time results provide confidence in CLASSIC's postdisturbance recovery and ability to estimate historical C fluxes Canada‐wide.

### 3.2 CLASSIC and Independent Reference Data Sets Capture a Mid‐20th to Early‐21st‐Century Carbon Sink

We simulated the C cycle in all of Canada from 1750 to 2023 using CLASSIC, driven by four disturbance scenarios. These scenarios reconstruct wildfire and harvest disturbance beginning in 1750 (Figures S4 and S5, Table S3) (Beaver et al. 2025). In early years, when observations are unavailable, the scenarios agree with broad historical patterns of fire Canada‐wide, simulated by an independent dynamic fire modeling approach driven by climatological drivers and population density (Figure S4e) (Curasi, Melton, Arora, et al. 2024; Curasi, Melton, Humphreys, et al. 2024). We evaluate the ensemble of simulations against an array of reference data sets (Appendix S1).

**FIGURE 3:**
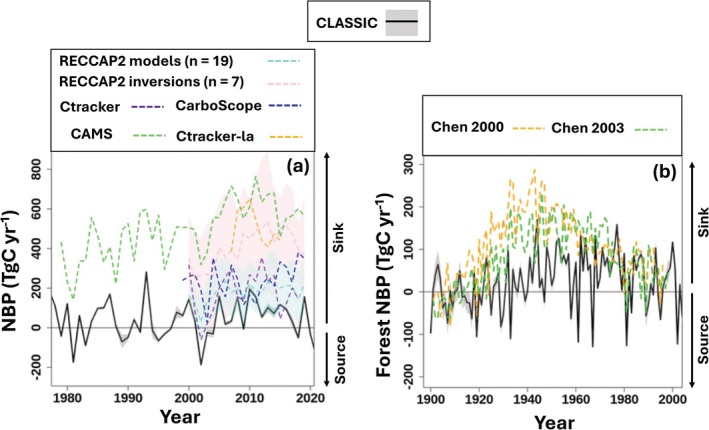
Comparisons between simulated NBP and reference data sets. This includes (a) Canada‐wide net biome productivity (NBP), and (b) NBP for forests only. A subset of each run is visualized for ease of comparison. The shaded region around each run is the minimum and maximum across the four ensemble members using different disturbance scenarios (Figure S5). For the Regional Carbon Cycle Assessment and Processes ensemble (RECCAP2), the mean and one standard deviation are visualized. See Figure S7 for a map of forested grid cells as defined by the Integrated Terrestrial Ecosystem Carbon model (InTEC).

CLASSIC's simulated net biome productivity (NBP, which encompasses the overall exchange of CO_2_ between terrestrial ecosystems and the atmosphere) reproduces the sign of the Canadian C sink in inversions (Figure 3a, 1980–2020). It also captures the broad temporal patterns of forest NBP from 1901 to 1996 in the InTec modeling framework (Chen, Chen, Liu, Cihlar, and Gray 2000; Chen, Chen, Liu, and Cihlar 2000; Chen et al. 2003) (Figure 3b and Table S1). Between 2007 and 2015, CLASSIC simulates Canada‐wide NBP between 85 and 112 Tg C year^−1^ (positive values denote a land sink), whereas individual inversions range from 220 to 638 Tg C year^−1^ (Figure 3a). Average NBP in an ensemble of seven inversions, from RECCAP2, is 458 (SD ±265 Tg C year^−1^) and 196 (SD ±131 Tg C year^−1^) from an ensemble of nineteen bottom‐up models. CLASSIC falls within one standard deviation of other models and within two standard deviations of the inversions. The smaller C sink in CLASSIC compared to inversions is due to higher respiratory fluxes in the winter and shoulder seasons (Figure S6). Unlike many LSMs, CLASSIC does not underestimate winter respiratory fluxes but rather may overestimate them (Arndt et al. 2023; Natali et al. 2019).

Despite a weaker C sink than inversions, CLASSIC is closer, in magnitude and temporal trends, to InTEC (Figure 3b). InTEC simulates NBP in Canadian forests aspatially (Chen, Chen, Liu, Cihlar, and Gray 2000; Chen, Chen, Liu, and Cihlar 2000) and, in further refinements, using a spatially explicit 1 × 1 km grid (Chen et al. 2003) (Figure S7). The spatially explicit version of InTEC adapts the Boreal Ecosystem Productivity simulator (Liu et al. 2002) to simulate daily variations in Net Primary Productivity (NPP) based on remotely sensed data. It then reconstructs historical NPP at an annual time step, simulates major C pools, and simulates NBP. It accounts for disturbance and nondisturbance forcings using empirical and semiempirical functions. Between 1940 and 1960, when the forest C sink peaks in InTEC, CLASSIC simulates a C sink (NBP = 44 to 68 Tg C year^−1^) in forests, albeit lower than in InTEC (NBP = 146 to 195 Tg C year^−1^; Figure 3b).

Comparing inversions and process‐based models in Canada must be done through a lens that accounts for fundamental differences in the underlying frameworks and structural and scale‐dependent complications. CLASSIC has the advantage of simulating NBP at the site level as well as Canada‐wide, in forested, nonforested, managed, and unmanaged portions of the landscape, allowing for direct comparisons of NBP estimates at different scales. There are many potential sources of difference between inversions and process‐based models. CO_2_ source sink estimated using global atmospheric CO_2_ inversion lose robustness at regional scales, particularly as Canada is discretized as only 1 to 5 regions in global systems (e.g., Rödenbeck et al. (2003); Deng et al. (2014)). Regional inversion systems use finer subdivisions (tens of regions or more; for example, Hu et al. (2019)), but their accuracy is limited by coarse‐resolution atmospheric transport models and a sparse network of CO_2_ observations across Canada. Inversions at the regional to country level exhibit wider variation between systems and between individual ensemble members (Figures 3a and S8) (Peylin et al. 2013). Other intercomparisons at the scale of North America show differences of similar magnitude with stronger sinks in inversions (Poulter et al. 2025). These differences may be due to uncertainty in anthropogenic CO_2_ emissions inventories used by inversions, given that a weaker northern hemisphere C sink is more consistent with remotely sensed changes in vegetation biomass (Randerson et al. 2025).

On the other hand, CLASSIC NBP may also be biased as it does not represent lateral fluxes, including the riverine flows of C from land to ocean, trade fluxes, and the transient impacts of deglaciation on peatland C sequestration (Harden et al. 1992; Poulter et al. 2025). Lateral fluxes through trade and riverine flow would decrease C transfers into soil and litter and decrease respiration (Ciais et al. 2021; Poulter et al. 2025). Approximately 25 TgC year^−1^ from litter and soil is exported to oceans via riverine transport, reducing terrestrial respiration, but this flux remains difficult to quantify due to spatial variability and measurement limitations (Poulter et al. 2025). Similarly, forest product fluxes are treated as a localized carbon source in CLASSIC NBP (Figure 5). Around 65% of harvested lumber was exported from Canada in 2025 (NRCAN 2026), with the quantity and fate of other harvested wood products remaining difficult to characterize. If some of the historical C uptake detected by inversions were due to the preindustrial soil C pool not in reality being in equilibrium (an assumption made by design in LSMs), we could expect a persistent and uniform NBP bias. CLASSIC, and other LSMs do not explicitly simulate slow but persistent peatland soil C accumulation (Harden et al. 1992; Smyth, Metsaranta, Tompalski, et al. 2024; Smyth, Metsaranta, Fortin, et al. 2024). Peatlands account for around 16–35 TgC year^−1^ of uptake (Harden et al. 1992; Roulet 2000). The extent to which this uptake is implicitly represented through the parameterization and the impact of this model's state bias on fluxes is unknown (Arora et al. 2023; Eyring et al. 2021; Flato et al. 2013; Knutti et al. 2010; Seiler et al. 2024). This range of uptake would, however, not account for the consistent bias between land surface models and inversions. Peatlands are estimated to make up ~32% (190 Pg) of Canada's soil C pool (Sothe et al. 2022). Which should be accounted for when using CLASSIC's wall‐to‐wall estimates (Table S4; Figure 5; Appendix S1). Explicitly representing peatlands is a key future step in tailoring CLASSIC to Canada, especially given the vulnerability of peatland C to climate change (Curasi et al. 2023; Roulet 2000; Sothe et al. 2022; Turetsky et al. 2010).

In InTEC, processes like forest age impacts on productivity are represented using semiempirical functions. In CLASSIC, recovery and NPP itself are mechanistically represented independent of remotely sensed data (Curasi, Melton, Arora, et al. 2024; Curasi, Melton, Humphreys, et al. 2024). That said, more detailed vegetation demography might better capture forest regeneration and the historical C sink better in CLASSIC (O'Sullivan et al. 2024; Pugh et al. 2019). CLASSIC does not explicitly simulate nutrient cycling and accounts for the absence of nutrient limitation through empirical downregulation (Arora et al. 2009). Therefore, it does not represent the impacts of changing anthropogenic nitrogen deposition (Chen, Chen, Liu, Cihlar, and Gray 2000; Chen, Chen, Liu, and Cihlar 2000; Chen et al. 2003; Propson et al. 2024). Nonetheless, CLASSIC captures the sign and presence of the C sink in Canada and the underlying processes, and can therefore synthesize the drivers of the historical C sink.

### 3.3 Early 20th‐Century Disturbance Modulates the Mid‐20th‐Century Carbon Sink

We investigate how different processes influenced the Canadian C sink in the mid‐20th century (1940–1960). This period is the peak of the forest C sink highlighted by InTEC and nadir of disturbance in the drivers (Figures S4 and S5) (Chen, Chen, Liu, Cihlar, and Gray 2000; Chen, Chen, Liu, and Cihlar 2000). When CLASSIC is driven with four disturbance scenarios (Figure S5), the model's average C sink varies in the mid‐20th century, due to disturbance in the early‐20th century (1900–1920; Figure S9). Greater early‐20th‐century disturbance drives a stronger C sink in the mid‐20th and early‐21st centuries (2002 to 2022; Figure S9).

**FIGURE 4:**
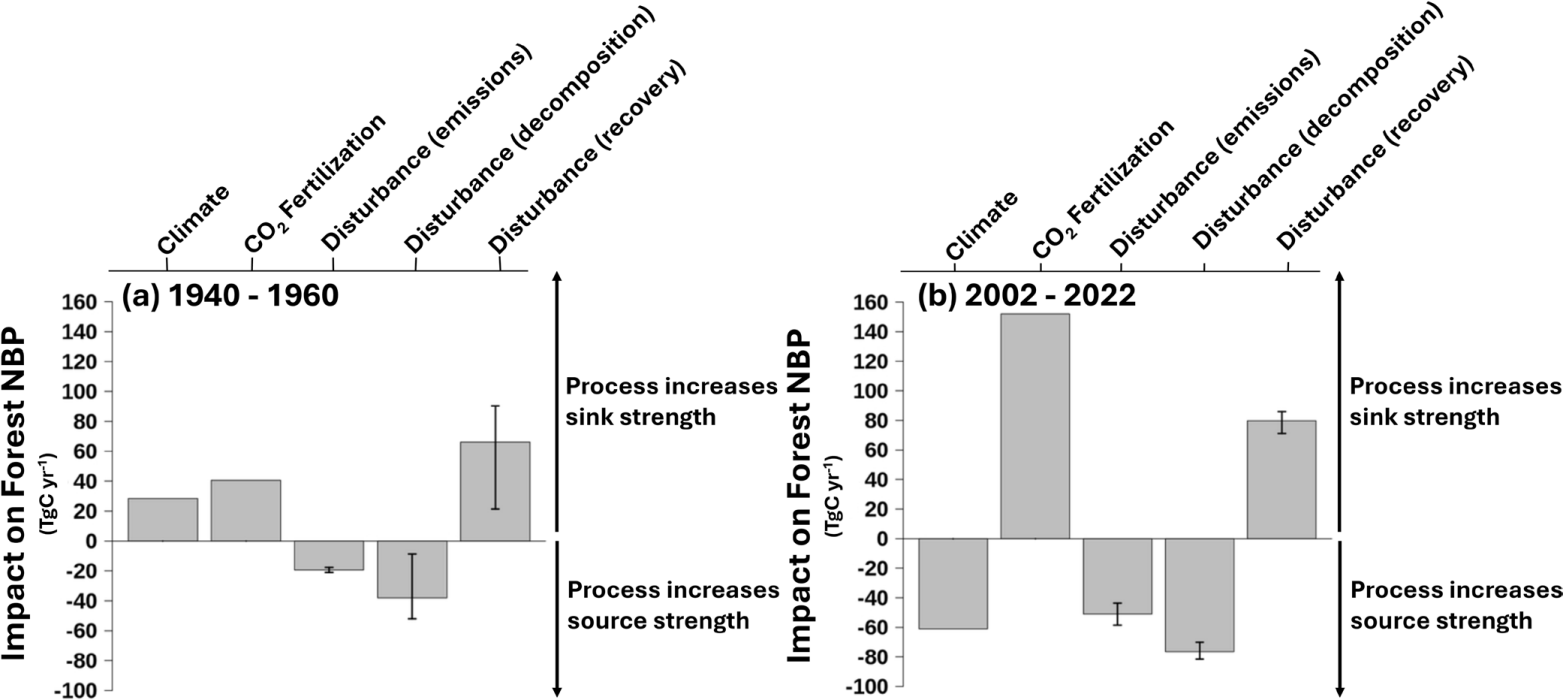
Impact of various forcings, including the lagged impact of disturbance before 1920, on the mid‐20th‐century (1940–1960) and early‐21st‐century (2002–2022) forest carbon sink. Factorial analysis that quantifies the impact of climate and CO_2_, the immediate impacts of disturbance, and the lag impact of disturbance due to decomposition and recovery in forested tiles on the change in average net biome productivity from (a) 1940–1960 and (b) 2002–2022. The error bars for disturbance processes show the maximum and minimum impacts of different disturbance forcings. See Figure S7 for a map of forested grid cells as defined by the Integrated Terrestrial Ecosystem Carbon model (InTEC).

Carrying out factorial runs with CLASSIC illustrates the impact of different processes driving the C sink (Figure 4a; Appendix S2). In the mid‐20th century (1940–1960), climate, CO_2_ fertilization, and vegetation recovery contributed a sink of 36, 52, and 85 Tg C year^−1^, respectively (Figure 4a). The sensitivity of NBP to CO_2_ fertilization is 1.2 Tg C year^−1^ per ppm atmospheric increase. Direct emissions by fire, timber harvest, and decomposition of litter after disturbances contribute a source of −25 and −49 Tg C year^−1^, respectively. The majority of the mid‐20th century C sink originates from disturbance recovery, where vegetation takes up C as stands regrow (Figure 4a).

The uncertainty range assigned to stands regeneration (NBP contribution = 21 to 90 Tg C year^−1^) stems from the multiple disturbance scenarios before 1918 (Figure 4a, Figures S4, S5, and S9). The scenarios span a range of competing assumptions about early‐20th‐century disturbance, including average and elevated disturbance frequencies (Figure S5). Elevated early‐20th‐century disturbance yields a stronger C sinks in the mid‐20th‐century, but this uncertainty diminishes by the early‐21st century (e.g., Figure 4 error bars; Figure S9). Capturing early‐20th‐century disturbance at any level is essential for simulating mid‐20th century and early‐21st century C uptake (e.g., factorial runs with no early‐20th‐century disturbance in Figure S9b).

The total area disturbed in this period is unknown due to a lack of records. Moreover, CLASSIC does not simulate nonstand replacing disturbance (i.e., insects, windthrow, selective logging), implying total disturbance across the simulation period is larger than recorded (Beaver et al. 2025; Chen, Chen, Liu, Cihlar, and Gray 2000; Chen, Chen, Liu, and Cihlar 2000; Kurz et al. 1995; Stocks et al. 2002; Van Wagner 1988). Before 1918, the scenarios agree with simulations from an independent dynamic wildfire modeling approach driven by climatological drivers and population density (Figure S4e) (Curasi, Melton, Arora, et al. 2024; Curasi, Melton, Humphreys, et al. 2024). There is strong anecdotal evidence that colonization, westward expansion, and industrialization led to increased disturbance from 1850 to 1920, which waned because of conservation initiatives and sustainable forest management (Drushka 2003; Parks et al. 2025). Ultimately, the net C sink in Canada's forests in the mid‐20th‐century is primarily a product of lagged disturbance impacts in the early‐20th‐century (1900–1920) (O'Sullivan et al. 2024; Pugh et al. 2019), alongside CO_2_ fertilization and climate effects.

### 3.4 Canada's Terrestrial C Cycle in the Early‐21st‐Century

In the early‐21st‐century (2000–2022), Canada's forests are being pushed toward a net source. CO_2_ fertilization is a strong sink, increasingly offset by disturbance and climate (Figure 4b). CO_2_ fertilization (152 Tg C year^−1^; Figure 4b) has a larger effect compared to the mid‐20th‐century (1940–1960), reflective of the large cumulative increases in atmospheric CO_2_ concentration. The sensitivity of NBP to CO_2_ fertilization (1.3 Tg C year^−1^ per ppm of atmospheric CO_2_ increase) is, however, similar. The CO_2_ fertilization C sink is partially compensated by a net C source due to climate (−61 Tg C year^−1^), which has increased respiration more than photosynthetic uptake. The sink from disturbance recovery is larger than in the mid‐20th‐century from higher disturbance rates (80 Tg C year^−1^ on average). The uncertainty bounds for disturbance recovery (71 to 86 Tg C year^−1^) are more constrained by precise disturbance observations (Figures S4 and S5). As total disturbed area increases in the early‐21st‐century, the C source due to instantaneous disturbance emissions and disturbance decomposition (−128 Tg C year^−1^ combined) offsets the sink associated with recovery. Disturbance contributes a net C source of −48 Tg C year^−1^ overall (Figure 4b). Burned area has increased in Canada as a result of climate change and will continue increasing in the future (Curasi, Melton, Arora, et al. 2024; Curasi, Melton, Humphreys, et al. 2024; Girardin and Mudelsee 2008; Hanes et al. 2019; Stocks et al. 2002). The C source‐sink dynamics of Canada's forests are strongly influenced by disturbances and will be shaped by them in the future.

**FIGURE 5:**
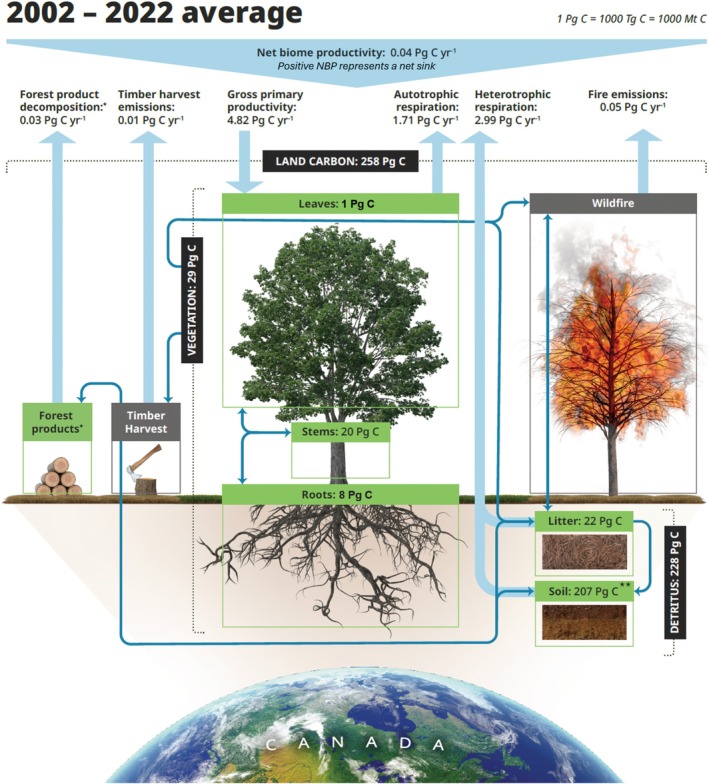
An overview of major carbon pools and fluxes within Canada in the early‐21st‐century (2002–2022). The mass of carbon held in each pool across all of Canada (i.e., forested and nonforested, managed and unmanaged land) is denoted in petagrams of carbon; major fluxes to and from the atmosphere are denoted as large blue arrows. Major pools and fluxes within the model are denoted as boxes and dashed lines; the diagram is simplified as compared to the underlying Canadian Land Surface Scheme Including Biogeochemical Cycles (CLASSIC) model for ease of visualization. *Note that forest product fluxes are not necessarily localized within Canada due to the export of forest products, but are simulated as being localized within Canada and captured in net biome productivity in the CLASSIC model (See Results & Discussion). **Note that CLASSIC does not yet explicitly represent the peatland carbon pool (~190 Pg; Table S4; Appendix S1; Results & Discussion).

**FIGURE 6:**
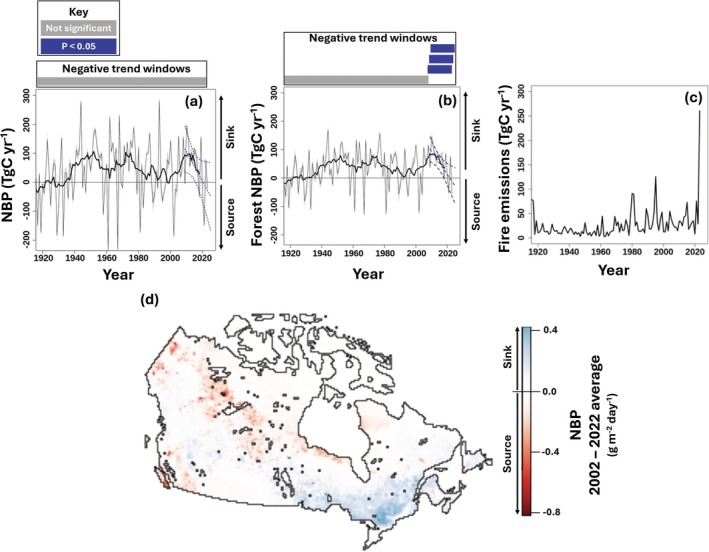
An overview of Canada's terrestrial C cycle. Including (a) net biome productivity (NBP) Canada‐wide, (b) Forest NBP, (c) wildfire emissions in the form of CO_2_, and (d) the spatial distribution of NBP Canada‐wide. In plots a and b, the thick solid lines are 10‐year running means. The blue lines show trends over the last 15 years to extend the moving average and include the associated 95% confidence intervals, with thicker dashes denoting significance. Significant 15‐year negative trend windows that would result in a change in the sign of the moving average are denoted above in “a” and “b”. See Figure S7 for a map of forested grid cells as defined by the Integrated Terrestrial Ecosystem Carbon model (InTEC).

Evaluating the C cycle using CLASSIC provides estimates of C fluxes and pools Canada‐wide (Figures 5 and S10). The Canadian land surface has been a net C sink, on average, from 1920 to 2023 (mean NBP = 44 Tg C year^−1^; Figure 6a). The primary contributor is forests (forest NBP = 38 Tg C year^−1^; Figure 6b). Nonetheless, Canada has been a C source in numerous individual years due to wildfire (Figure 6c). 2023 stands out with record burned area (12.6–15.2 Mha) and emissions of 210 to 259 Tg C year^−1^ (equivalent to 770 to 949 Tg CO_2_ year^−1^). Despite the wildfire emissions, vegetation GPP peaks in 2023 at 5.5 Tg C year^−1^ (Figure S11), offsetting a significant portion of fire emissions (Figure 6a,b) (Dong et al. 2026). Over the last 15 years Canada‐wide NBP is not definitively trending towards the source sink transition line (slope across 2009–2023 = −10.2 Tg C year^−1^; *p* = 0.06; Figure 6a). However, forests are trending towards being a C source (slope across 2009–2023 = −8.1 Tg C year^−1^; *p* = 0.01; Figure 6b). Testing the entire time series for negative trends using 15 year regression windows suggests this trend is unprecedented over the last ~100 years (Figure 6b). It is primarily driven by wildfire disturbance concentrated in central Canadian forests where lower GPP slows vegetation recovery (Figure 6d, S3c, and S12) (Coops et al. 2018; Pelletier et al. 2024).

## 4 Discussion

Across scales, top‐down and bottom‐up methods broadly agree on a net C sink in Canada, driven by the recovery of vegetation from disturbance and CO_2_ fertilization. CLASSIC includes a detailed representation of disturbance and thus simulates a weaker sink within one standard deviation of other models and within two standard deviations of inversions. Differences among existing studies arise from their different spatial and temporal scales, forcing inputs, and associated uncertainties (Dietze 2017; Marchand et al. 2018). CLASSIC aligns with the ecology of Canada's forests, where leaf to individual tree level observations suggest nutrient limitation and drought stress constrain vegetation's response to CO_2_ fertilization and warming (Figure 4b) (Hickler et al. 2008; Lesven et al. 2024; Norby and Zak 2011). CLASSIC is consistent with observations in individual trees and dendrochronological studies (Lesven et al. 2024). These observations would be less likely to capture a sink driven by landscape‐scale vegetation turnover and recovery far in the past (Figures 4a and S9) (Girardin et al. 2016; Marchand et al. 2018). The empirical down‐regulation scheme that represents progressive nutrient limitation matches V_cmax_ down‐regulation observed experimentally (18% in Bigras and Bertrand (2006); see also table 2 in Arora et al. (2009) and *CLASSIC* methods). Limited experimental data points to V_cmax_ downregulation in seedlings under CO_2_ enrichment (Johnsen and Seiler 1996), with greater downregulation in adult trees (Dusenge et al. 2024). Observational studies in adult trees do not consistently demonstrate increased growth due to CO_2_ in boreal regions (Girardin et al. 2016; Price et al. 2013) or at global scales (Gedalof and Berg 2010) except for studies in western Canada in recent years (Messaoud and Chen 2011). Experiment manipulations like FACE are more likely to capture evidence of nutrient limitation and acclimation than historical observations like FLUXNET (Raoult et al. 2024). Further experimental and observational evidence is critical to constraining CO_2_ fertilization, nutrient limitation and interactions with warming and drought in Canadian ecosystems, particularly when modeling high warming, high atmospheric CO_2_ future scenarios. Our results also show that global disturbance history and its demographic impacts should be accounted for in LSMs. These processes are key to realistically simulating Canada's 20th‐century C sink (Fisher and Koven 2020; Pongratz et al. 2018). In addition, transient impacts due to the initial state of the land surface mediated by past disturbance (e.g., Harden et al. (1992)) can influence fluxes on the landscape.

Since 2009, the immediate impacts of disturbance have begun to overwhelm the recovery‐driven C sink in Canadian forests. The component processes driving NBP have been amplified. The impacts of disturbance and CO_2_ fertilization have tripled since the mid‐20th century (Figure 4). Wildfire disturbance is increasing with climate change and will continue increasing under unmitigated climate change (Curasi, Melton, Arora, et al. 2024; Curasi, Melton, Humphreys, et al. 2024; Girardin and Mudelsee 2008; Hanes et al. 2019; Kirchmeier‐Young et al. 2024; Stocks et al. 2002). Destabilizing (positive) feedbacks, whereby fire alters vegetation promoting intense future fires, land cover change, and disrupts vegetation recovery, could contribute to this trend (Parks et al. 2025). Extending the modelled moving average NBP over the last 15 years suggests that Canadian forests crossed the source‐sink transition around 2021 (SD ±2.6 years). Projected increases in fire suggest that the trend towards source status could persist (Curasi, Melton, Arora, et al. 2024; Curasi, Melton, Humphreys, et al. 2024). Increased future disturbance would lead to additional emissions, rising wildfire and forestry management costs, and imperil efforts to manage the C sink through sustainable land management practices and fire suppression.

## Author Contributions

**Alex J. Cannon:** writing – review and editing, data curation. **Vivek K. Arora:** writing – review and editing. **Michael A. Wulder:** writing – review and editing, data curation. **Txomin Hermosilla:** writing – review and editing, data curation. **Elyn R. Humphreys:** writing – review and editing, conceptualization, funding acquisition, resources. **Sung‐Ching Lee:** writing – review and editing, data curation. **Joe R. Melton:** writing – review and editing, funding acquisition, resources, conceptualization. **Jason Beaver:** writing – review and editing, data curation. **Jing M. Chen:** writing – review and editing, data curation. **Salvatore R. Curasi:** writing – review and editing, conceptualization, methodology, software, formal analysis, writing – original draft, validation, visualization.

## Funding

This work was supported by Natural Sciences and Engineering Research Council of Canada, ALLRP 556430‐2020.

## Conflicts of Interest

The authors declare no conflicts of interest.

## Supporting information


**Appendix S1:** The ensemble of CLASSIC runs, averaged over 2000 to 2013, captures the spatial distribution of above ground biomass (AGB; CLASSIC = 3.9−4.0 reference = 1.9−5.5 kg C m^2^) soil C (CSOIL; CLASSIC = 23.0−23.1; reference = 13.7−45.5 kg C m^2^), and gross primary productivity (GPP; CLASSIC = 1.536−1.541 reference = 1.12−1.54 gC m^2^ day^−1^) across Canada.
**Appendix S2:** We investigate the relative impacts of different processes on the Canadian carbon sink in different periods using factorial analysis.
**Figure S1:** Modelled versus observed plots for site‐level simulations of wildfire and harvest disturbance.
**Figure S2:** Observed fluxes and biomass for a chronosequence of sites across Canada compared to CLASSIC without wildfire and harvest disturbance (counterfactual).
**Figure S3:** Comparisons between Canada‐wide classic simulations and gridded reference data sets, including (a) above‐ground biomass, (b) soil carbon, (c) gross primary productivity, and (d) fire emissions.
**Figure S4:** Drivers for CLASSIC. Canada‐wide mean summary plots including (a) mean annual temperature, (b) mean annual precipitation rate, (c) atmospheric CO_2_ concentration, (d) total harvest land area, and (e) total burned land area.
**Figure S5:** Drivers for CLASSIC. Canada‐wide mean summary plots of total disturbed area.
**Figure S6:** Plot comparing monthly average NBP between 2006 and 2015 from four CLASSIC (See Table S3, run #1–4) to an ensemble of inversions (See Table S1).
**Figure S7:** Forested grid cells on the CLASSIC model grid in Canada as defined by InTec.
**Figure S8:** Plots visualizing the 18 ensemble members composing CarbonTracker‐Lagrange.
**Figure S9:** Plot showing the lag effect of disturbance in the early 20th century (1900–1920) on average forest NBP in the (a) mid 20th century (1940–1960) and (b) early 21st century (2002–2022).
**Figure S10:** An overview of Canada's major carbon pools and fluxes in the mid‐20th century (1940–1960).
**Figure S11:** Plots of (a) gross primary productivity and (b) ecosystem respiration Canada‐wide from four CLASSIC runs (See Table S3, run #1–4).
**Figure S12:** The spatial distribution of gross primary productivity, ecosystem respiration, and vegetation biomass Canada‐wide averaged from 2002 to 2022 from four CLASSIC runs (See Table S3, run #1–4).
**Table S1:** Overview of independent data sets.
**Table S2:** Detailed setup and other run information for FLUXNET sites included in the site‐level comparison suite.
**Table S3:** Overview of CLASSIC model runs.
**Table S4:** Comparisons between wall‐to‐wall carbon pool estimates from CLASSIC.

## Data Availability

The CLASSIC source code and data that support this publication are archived on Zenodo at https://doi.org/10.5281/zenodo.17556342.
