# Supplementary material for: Canada's Forests Are Shifting From a Recovery‐Driven Carbon Sink to a Disturbance‐Driven Carbon Source
Source: Glob Chang Biol. 2026 Jun 5;32(6):e70958. doi: 10.1111/gcb.70958 (PMC13240602; doi:10.1111/gcb.70958)
Supplement: Supplementary file 1 — Appendix S1: The ensemble of CLASSIC runs, averaged over 2000 to 2013, captures the spatial distribution of above ground biomass (AGB; CLASSIC = 3.9−4.0 reference = 1.9−5.5 kg C m2) soil C (CSOIL; CLASSIC = 23.0−23.1; reference = 13.7−45.5 kg C m2), and gross primary productivity (GPP; CLASSIC = 1.536−1.541 reference = 1.12−1.54 gC m2 day−1) across Canada. Appendix S2: We investigate the relative impacts of different processes on the Canadian carbon sink in different periods using factorial analysis. Figure S1: Modelled versus observed plots for site‐level simulations of wildfire and harvest disturbance. Figure S2: Observed fluxes and biomass for a chronosequence of sites across Canada compared to CLASSIC without wildfire and harvest disturbance (counterfactual). Figure S3: Comparisons between Canada‐wide classic simulations and gridded reference data sets, including (a) above‐ground biomass, (b) soil carbon, (c) gross primary productivity, and (d) fire emissions. Figure S4: Drivers for CLASSIC. Canada‐wide mean summary plots including (a) mean annual temperature, (b) mean annual precipitation rate, (c) atmospheric CO2 concentration, (d) total harvest land area, and (e) total burned land area. Figure S5: Drivers for CLASSIC. Canada‐wide mean summary plots of total disturbed area. Figure S6: Plot comparing monthly average NBP between 2006 and 2015 from four CLASSIC (See Table S3, run #1–4) to an ensemble of inversions (See Table S1). Figure S7: Forested grid cells on the CLASSIC model grid in Canada as defined by InTec. Figure S8: Plots visualizing the 18 ensemble members composing CarbonTracker‐Lagrange. Figure S9: Plot showing the lag effect of disturbance in the early 20th century (1900–1920) on average forest NBP in the (a) mid 20th century (1940–1960) and (b) early 21st century (2002–2022). Figure S10: An overview of Canada's major carbon pools and fluxes in the mid‐20th century (1940–1960). Figure S11: Plots of (a) gross primary productivity and (b) ecosystem r [file GCB-32-e70958-s001.pdf]

Supplementary information for: Canada's forests are shifting from a recovery-driven carbon sink to a disturbance-driven carbon source.

Salvatore. R Curasi[1,*,https://orcid.org/0000-0002-4534-3344], Joe R. Melton[2,https://orcid.org/0000-0002-9414-064X], Elyn R. Humphreys[3,https://orcid.org/0000-0002-5397-2802], Vivek K. Arora[1,https://orcid.org/0000-0002-2620-9342], Jason Beaver[4], Alex J. Cannon[2,https://orcid.org/0000-0002-8025-3790], Jing M. Chen[5], Txomin Hermosilla[6,https://orcid.org/0000-0002-5445-0360], Sung-Ching Lee[7,https://orcid.org/0000-0002-2615-2040] , Michael A. Wulder[6,https://orcid.org/0000-0002-6942-1896]

[1]Canadian Centre for Climate Modelling and Analysis, Environment and Climate Change Canada, Victoria, B.C., Canada
[2]Climate Research Division, Environment, and Climate Change Canada, Victoria, BC, Canada
[3]Department of Geography & Environmental Studies, Carleton University, Ottawa, ON, Canada
[4]National Wildlife Research Centre, Environment and Climate Change, Ottawa, ON, Canada
[5]Department of Geography, University of Toronto, Toronto, Ontario, Canada
[6]Canadian Forest Service (Pacific Forestry Centre), Natural Resources Canada, Victoria, BC, Canada
[7]Department of Biogeochemical Integration, Max Planck Institute for Biogeochemistry, Jena, Germany

*correspondence: Sal.Curasi@ec.gc.ca

Contents of this file

- Appendix S1 - S2 (including equations S1 - S6)
- Figures S1 - S12
- Tables S1 - S4
- References

Appendix S1:

The ensemble of CLASSIC runs, averaged over 2000 to 2013, captures the spatial distribution of above ground biomass (AGB; CLASSIC = 3.9 - 4.0 reference = 1.9 - 5.5 kg C m^{-2}) soil C (CSOIL; CLASSIC = 23.0 - 23.1; reference = 13.7 - 45.5 kg C m^{-2}), and gross primary productivity (GPP; CLASSIC = 1.536 - 1.541 reference = 1.12 - 1.54 gC m^{-2} day^{-1}) across Canada. For most of the landscape, CLASSIC falls near the center of the range of the four AGB reference datasets, three CSOIL reference datasets, and four GPP reference datasets (Fig. S3a-d; Table S1). There is a negative bias in CSOIL between 50 and 60 degrees as compared to the Sothe and SoilGrids data sets, but not the HWSD data (Fig S3b). This is likely because these data sets capture peatland soil carbon, which is not explicitly represented in CLASSIC [1,2]. This bias should be considered and potentially accounted for by using a separate product, e.g., Sothe et al. [2], when needed, when using the CLASSIC wall-to-wall estimates.

Fire emissions and modelled net biome productivity (NBP) Canada-wide show greater variability among the CLASSIC ensemble members because they are more significantly impacted by the disturbance forcings used to drive the model (Fig. S3d). Despite this, CLASSICs modelled fire emissions fall well within the range of four independent reference data sets (2003 - 2014 average CLASSIC = 30 - 43; reference = 21 - 45 Tg C $year^{-1}$; Fig. S3d). This is in line with CLASSIC and other LSMs exhibiting weak correlations between the model state variables (i.e., the size of the C pools) and net C fluxes (i.e., land C uptake over the historical period)[3–5]. This is because processes like disturbance, climate warming, and CO_2 fertilization primarily determine the latter. CLASSIC captures burn severity—the proportion of vegetation burned during fire—using a fixed parameterization that assumes the burned area drivers represent stand-replacing fire events. Different disturbance data sets may have different detection thresholds for stand-replacing disturbance due to the resolution of the underlying input data (i.e., satellite imagery) and the algorithms used. For example, vector data can have coarser delineation around water features and areas of non-stand-replacing disturbance, yet in this configuration of CLASSIC, fire is parameterized as being entirely stand-replacing[6]. This model parameterization may be more ecologically consistent with the detection thresholds realized by the raster-based disturbance data sets. Model runs using NFIS-based disturbance drivers may be more suitable for investigating the absolute magnitude of net C flux Canada-wide. Nonetheless, we average the entire ensemble of runs (See Table S3, runs #1 - 4) in synthesizing carbon fluxes and pools across Canada.

Appendix S2:

We investigate the relative impacts of different processes on the Canadian carbon sink in different periods using factorial analysis. The impact of climate on NBP is quantified using a run with transient climate alone ($NBP_{climate}$; simulation #5 in Table S3; Eqn. S1).

$$Climate = NBP_{climate} \quad \text{(S1)}$$

The impact of CO_2 fertilization is quantified as the difference between the runs with transient climate alone and a run with transient climate and transient atmospheric CO_2 concentrations ($NBP_{climate,CO2}$; simulations #6 in Table S3; Eqn. S2).

$$CO_2 = NBP_{climate,\, CO_2} - NBP_{climate} \quad \text{(S2)}$$

The immediate impacts of disturbance are quantified using the modelled disturbance emissions to the atmosphere for four runs that include transient climate, transient atmospheric CO_2 concentrations, and transient disturbance ($Emissions_{climate,CO2,disturbance}$; simulations #1 - 4 in Table S3; Eqn. S3).

$$Disturbance = -Emissions_{climate,\, CO_2,disturbance} \quad \text{(S3)}$$

The lagged decomposition resulting from the disturbance is quantified as a function that also includes four runs, where the disturbance is zeroed during the years being analyzed (1940 - 1960 in Fig. 4a and 2002 - 2022 in Fig. 4b; $NBP_{climate,CO2,\text{ no disturbance after yr.}}$; simulations #7 - 14 in Table S3; Eqn. S4). It also uses four runs that include transient climate, transient atmospheric CO_2 concentrations, and transient disturbance ($NBP_{climate,CO2,disturbance}$; simulations #1 - 4 in Table S3).

$$Post\ disturbance\ decomposition = NBP_{climate,\, CO_2,disturbance} - NBP_{climate,\, CO_2,no\ disturbance\ after\ yr.} + Emissions_{climate,\, CO_2,disturbance} \quad \text{(S4)}$$

Finally, the impact of disturbance recovery is quantified as a function that includes a run where the disturbance is zeroed in the years before the years being analyzed (before 1940 in Fig. 4a and before 2002 in Fig. 4b; $NBP_{climate,CO2,\text{ no disturbance before yr.}}$; simulations #15 - 18 in Table S3; Eqn. S5).

$$\begin{aligned} Regrowth = & \left(-Emissions_{climate,\, CO_2,disturbance} - NBP_{climate,\, CO_2,no\ disturbance\ before\ yr.}\right) \\ & + \left(\left(NBP_{climate,\, CO_2,no\ disturbance\ before\ yr.} - NBP_{climate,\, CO_2}\right) \right. \\ & \left. - \left(NBP_{climate,\, CO_2,disturbance} - NBP_{climate,\, CO_2,no\ disturbance\ after\ yr.}\right)\right) \end{aligned} \quad \text{(S5)}$$

We can validate the closure of this framework given that the individual NBP components capture the NBP of a standard CLASSIC run with all transient forcings (Eqn. S6).

$$\begin{aligned} & (Climate + CO_2 + Disturbance + Post\ disturbance\ decomposition + Regrowth) \\ & \quad - NBP_{climate,\, CO_2,disturbance} \approx 0 \end{aligned} \quad \text{(S6)}$$

Figures:

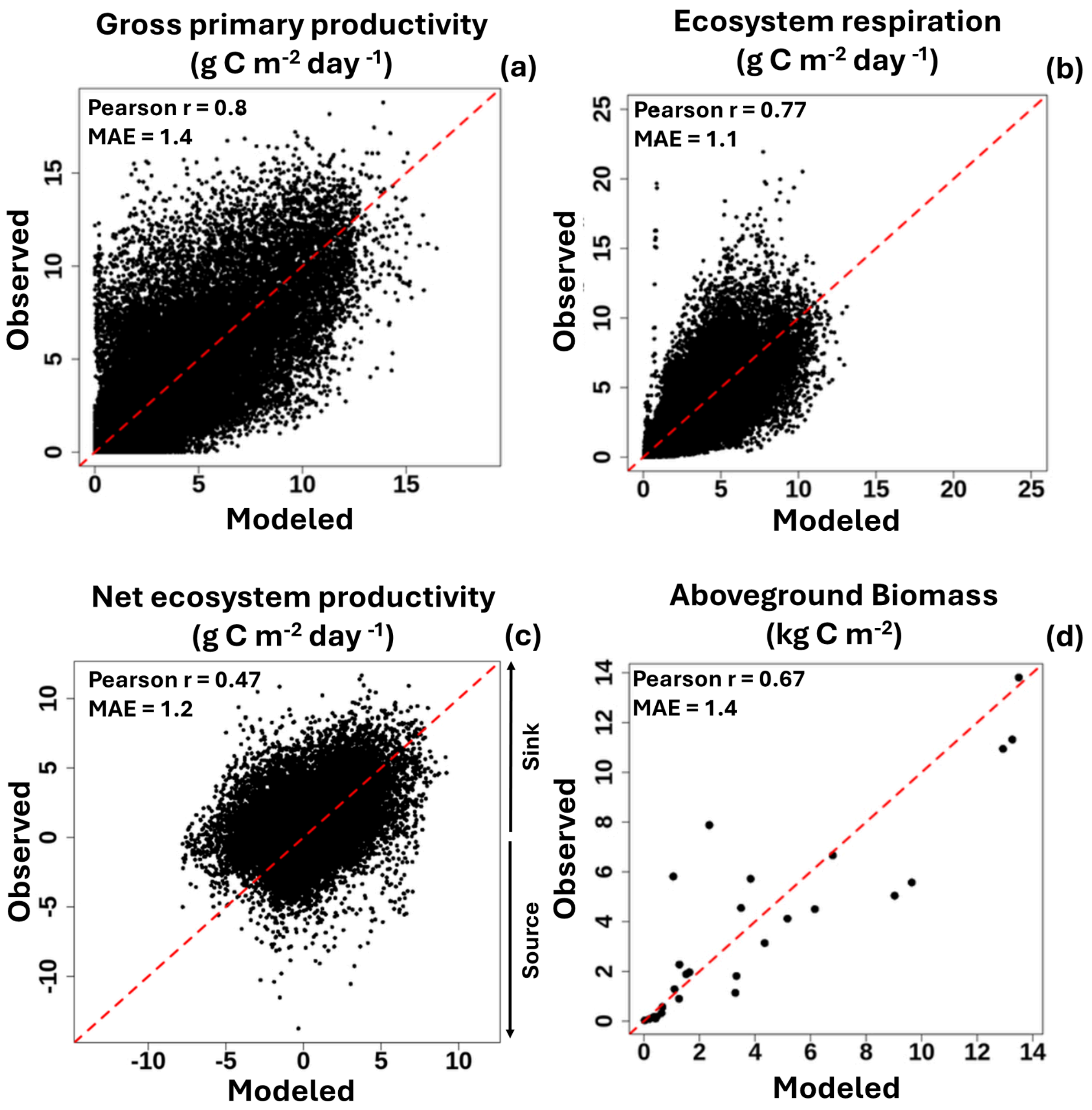

Figure S1: Modelled versus observed plots for site-level simulations of wildfire and harvest disturbance. Daily observed **a)** gross primary productivity, **b)** ecosystem respiration, and **c)** net ecosystem productivity from eddy flux towers compared to modelled outputs from CLASSIC (number of sites = 26; number of observations = 50,238). **d)** Annual above-ground biomass observed in site-level inventories compared to modelled outputs from CLASSIC (number of sites = 21; number of observations = 27). All panels include a red 1:1 line.

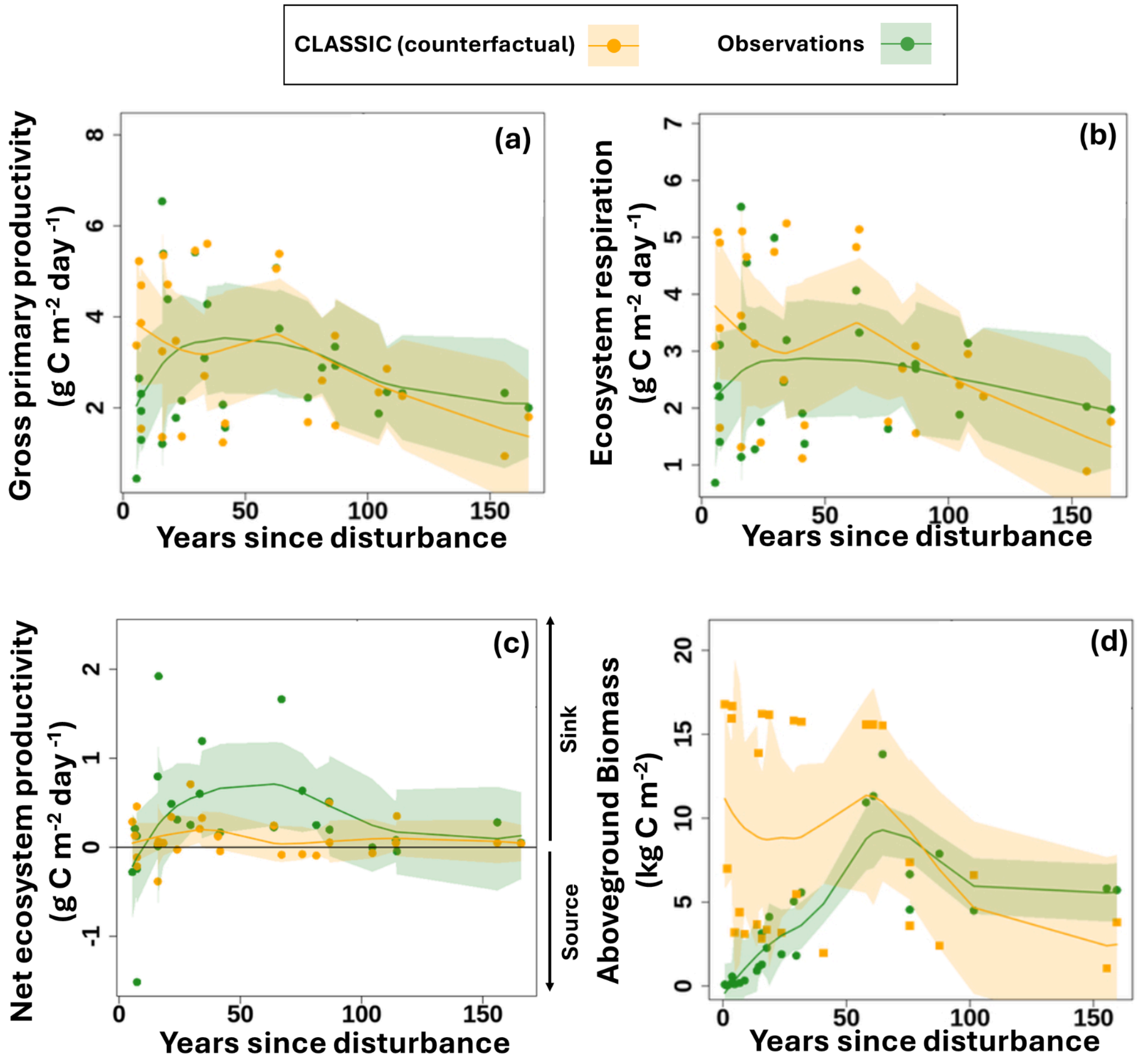

Figure S2: Observed fluxes and biomass for a chronosequence of sites across Canada compared to CLASSIC without wildfire and harvest disturbance (counterfactual). Average observed **a)** gross primary productivity, **b)** ecosystem respiration, and **c)** net ecosystem productivity from eddy flux towers (number of sites = 26), as well as **d)** above-ground biomass from site-level inventories (number of sites = 21). The observations are plotted against the number of years since disturbance at the site alongside counterfactual simulations from CLASSIC (i.e., simulations without disturbance). Each point represents the average for an individual site summarised using loess smoothed regression lines with shaded 95% confidence intervals.

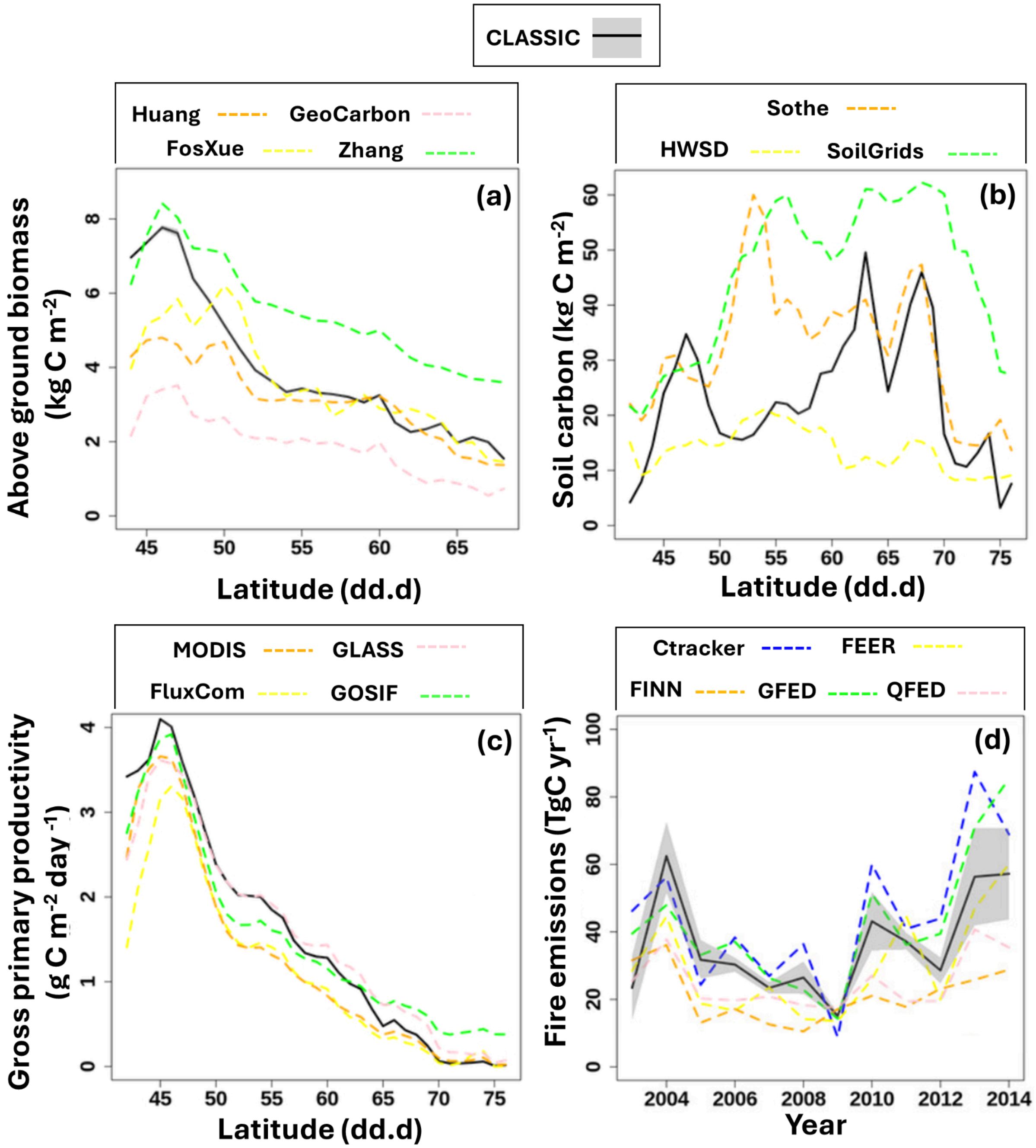

Figure S3: Comparisons between Canada-wide classic simulations and gridded reference data sets, including **a)** above-ground biomass, **b)** soil carbon, **c)** gross primary productivity, and **d)** fire emissions. The shaded region is the minimum and maximum of the four ensemble members.

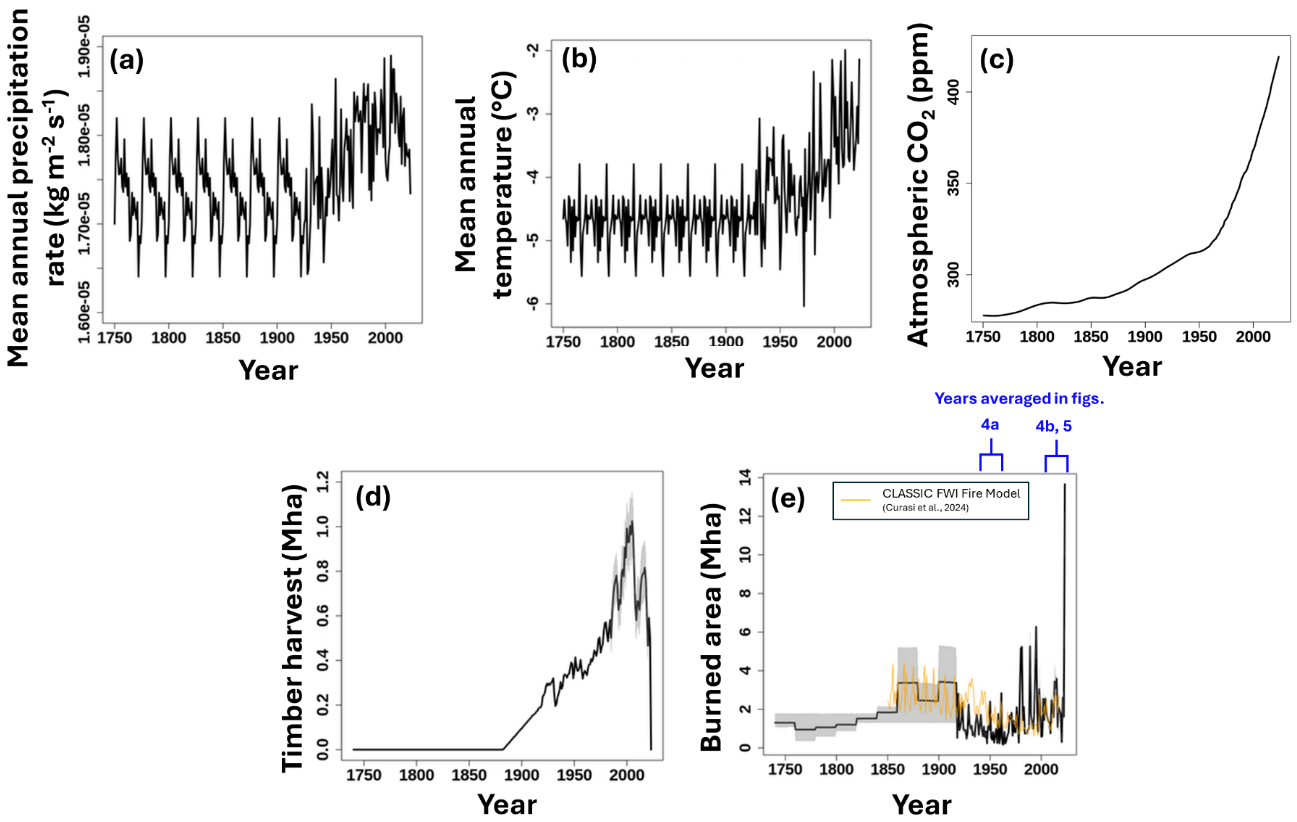

Figure S4: Drivers for CLASSIC. Canada-wide mean summary plots including a) mean annual temperature, **b)** mean annual precipitation rate, **c)** atmospheric CO_2 concentration, **d)** total harvest land area, and **e)** total burned land area. Plots a and b are the annual mean of the CRU model driver; the repeating patterns are the climate loop used in the early years when reanalysis is unavailable. Plots d and e summarize four disturbance forcings, and the shaded region represents the minimum and maximum annual values among the forcings. The burned area figure denotes the time slices analyzed in Figures 4, 5, and S5. Panel "e" includes a trace from CLASSIC's FWI-based wildfire model over the historical period; that model is not used herein, but shown as a point of comparison for patterns of fire in the distant past. See Curasi et al.,[7] for associated equations and model configuration details.

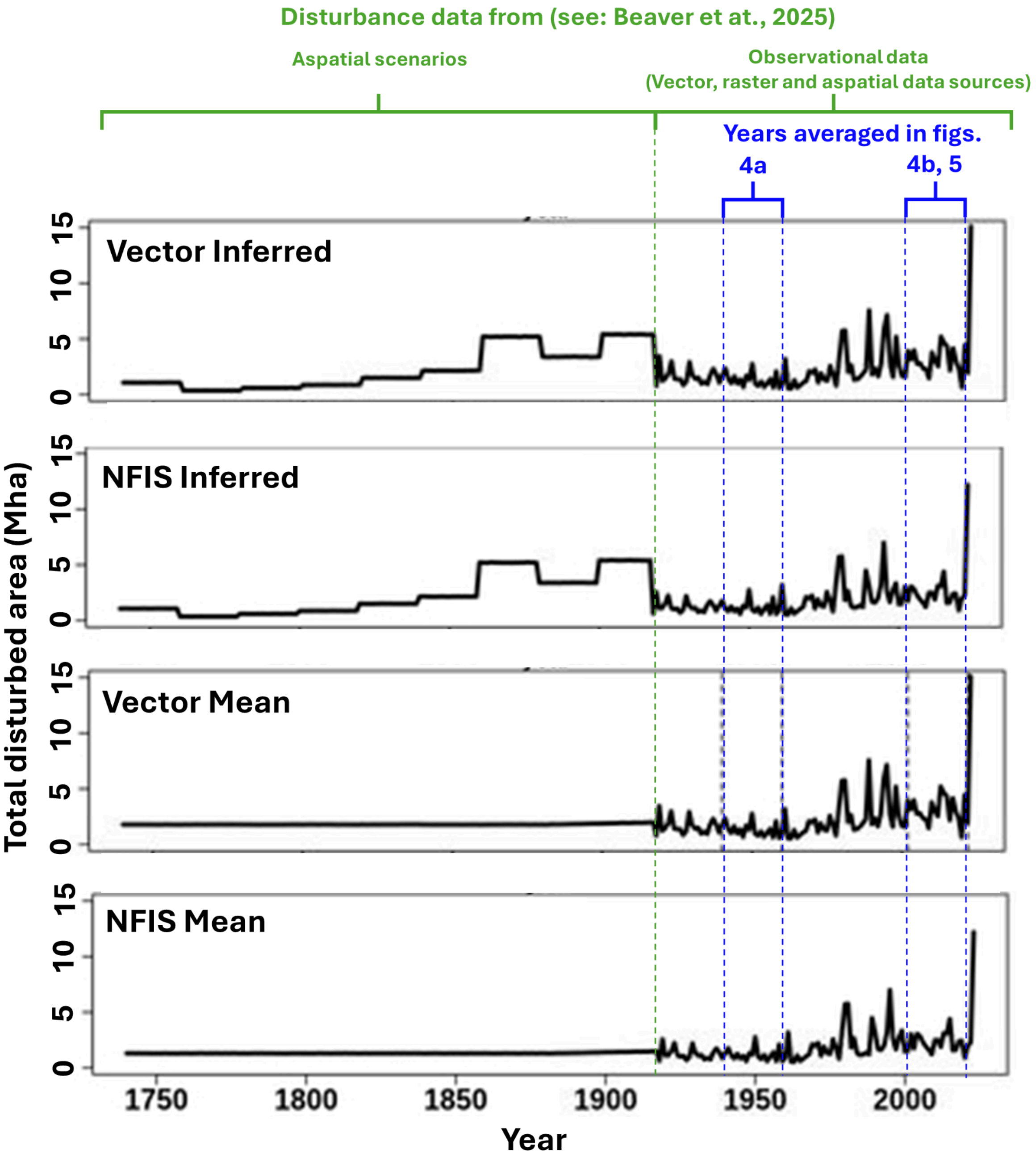

Figure S5: Drivers for CLASSIC. Canada-wide mean summary plots of total disturbed area. The disturbance forcings shown in S4d-e and documented in Table S3 are shown here individually.

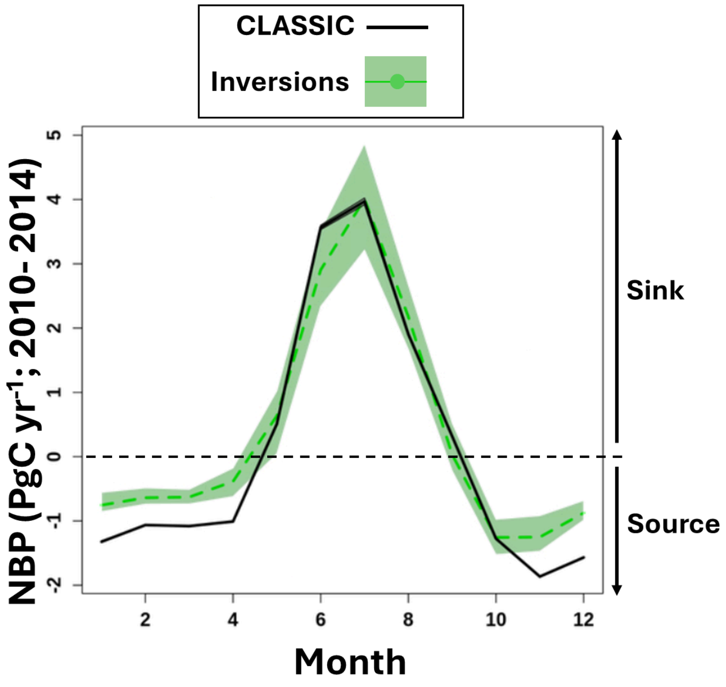

Figure S6: Plot comparing monthly average NBP between 2006 and 2015 from four CLASSIC runs (See Table S3, run #1 - 4) to an ensemble of inversions (See Table S1). The shaded regions represent the minimum and maximum monthly average NBP (Table S1; note that in CLASSIC, there is limited variation in these quantities when runs are averaged monthly).

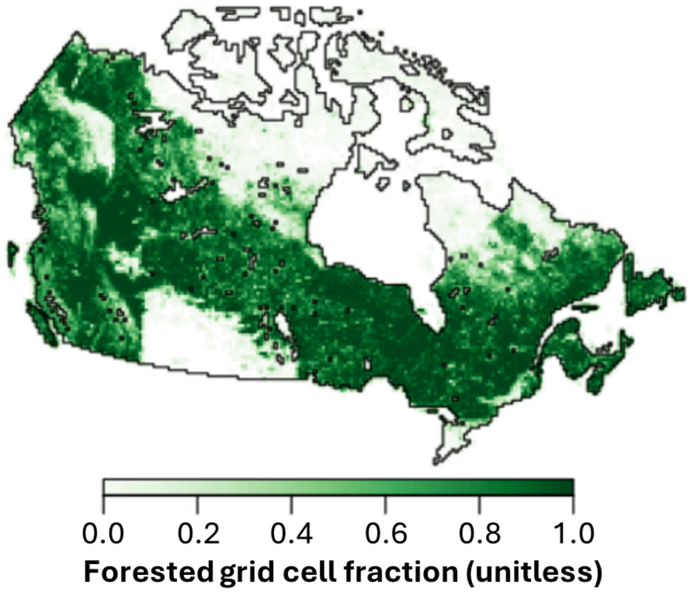

Figure S7: Forested grid cells on the CLASSIC model grid in Canada as defined by InTec[8]. InTec uses a 1x1 km grid. Because the resolution of InTec is higher than that of CLASSIC, fractional weights are calculated and shown near the forest's margins.

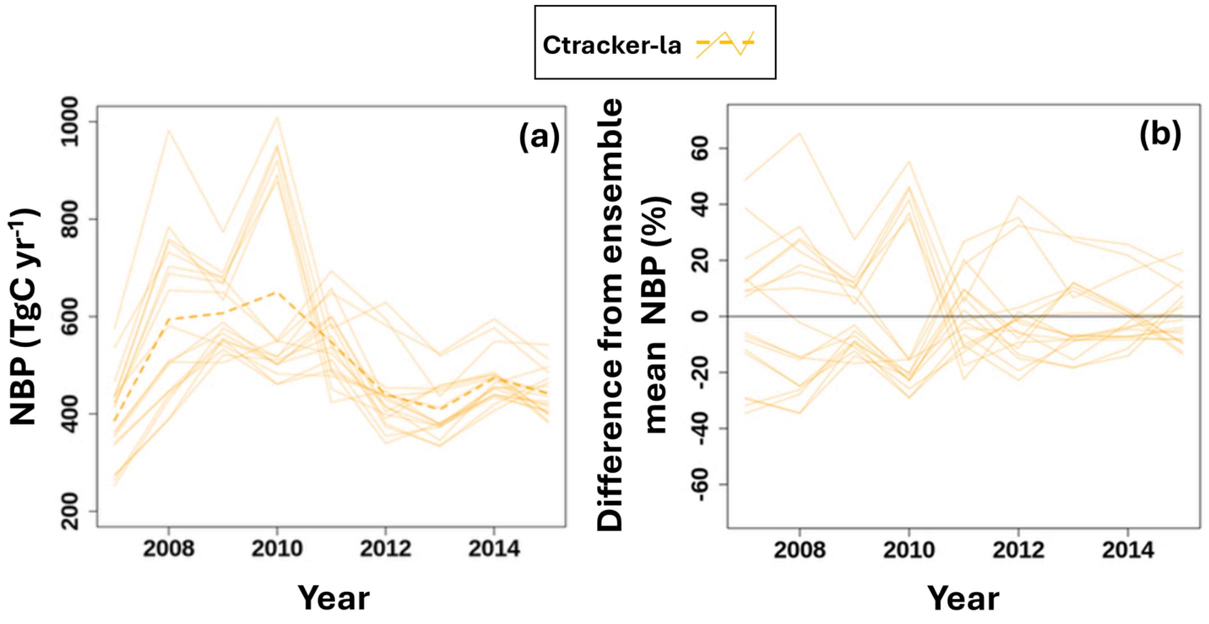

Figure S8: Plots visualizing the 18 ensemble members composing CarbonTracker-Lagrange

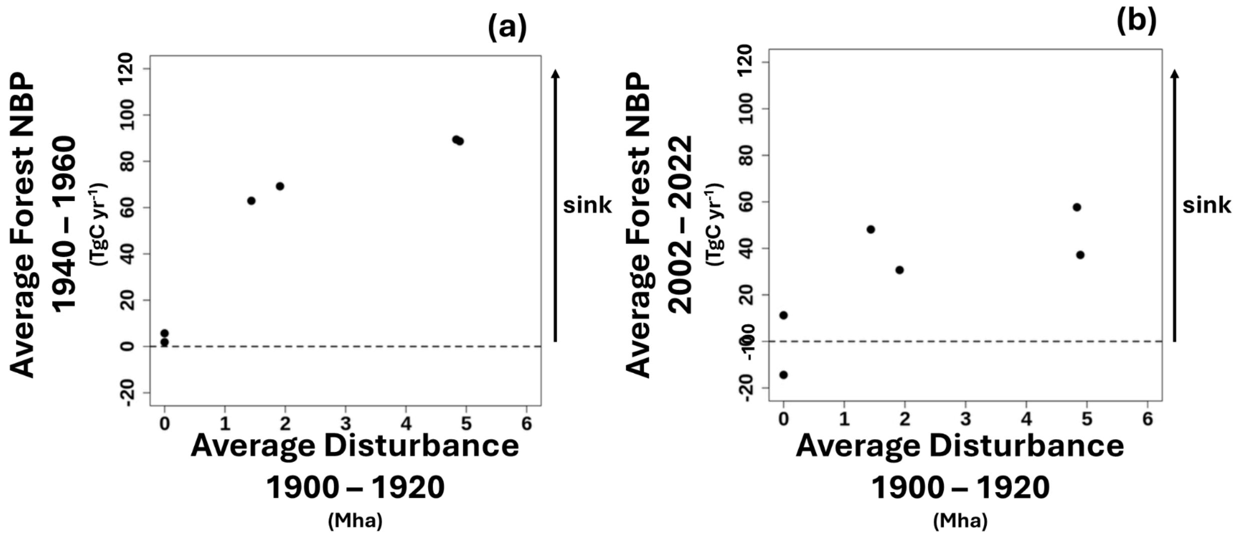

Figure S9: Plot showing the lag effect of disturbance in the early 20th century (1900 - 1920) on average forest NBP in the **a)** mid 20th century (1940 - 1960) and **b)** early 21st century (2002 - 2022). For illustrative purposes, factorial runs with no disturbance before 1940 (i.e., runs 15 - 16 in Table S3) are visualized in addition to the four main disturbance scenarios analyzed in this study (i.e., runs 1 - 4 in Table S3 that use the scenarios shown in Fig. S5).

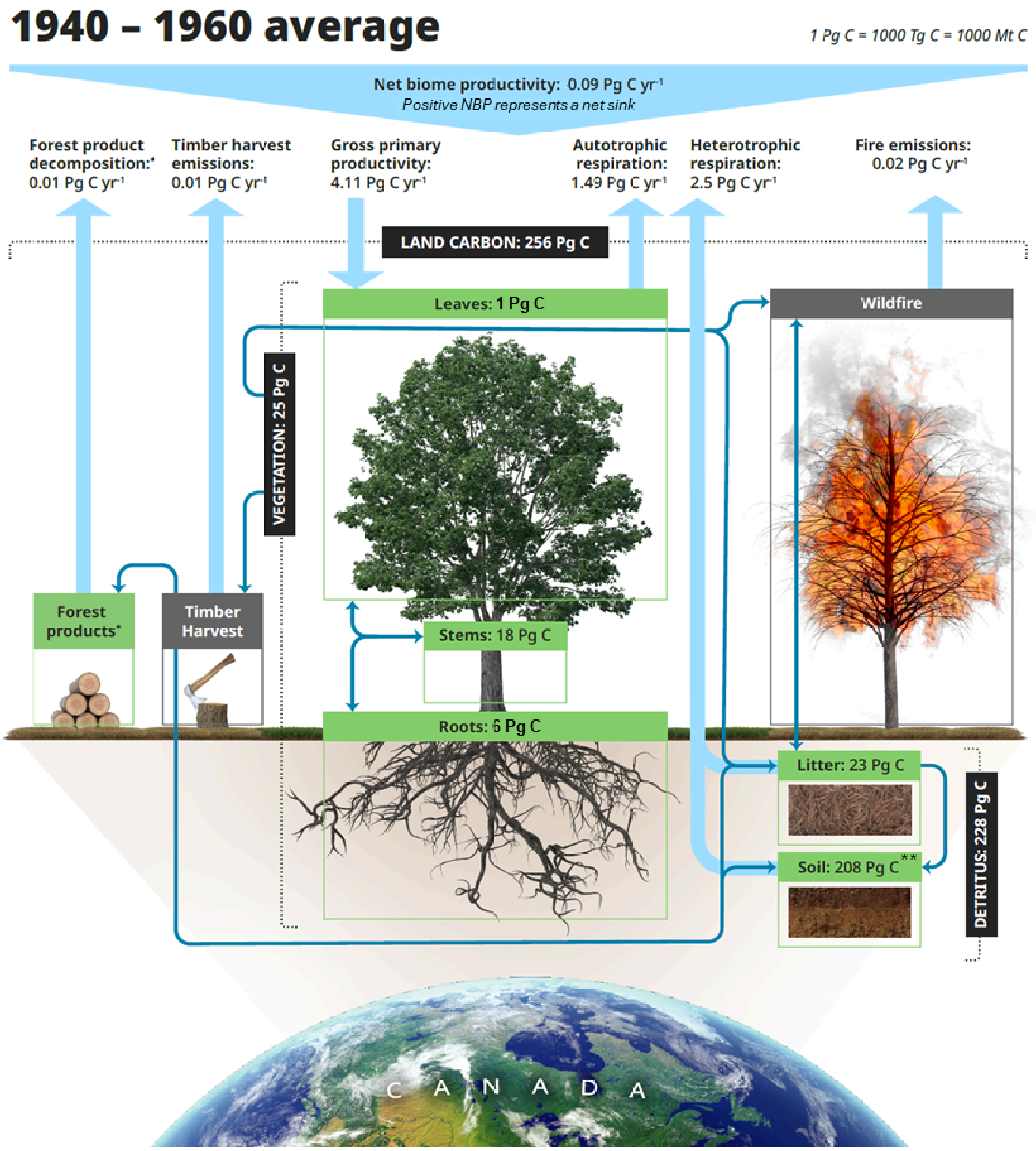

Figure S10: An overview of Canada's major carbon pools and fluxes in the mid-20th century (1940 - 1960). The mass of carbon held in each pool is denoted in petagrams of carbon; major fluxes to and from the atmosphere are denoted as large blue arrows. Major pools and fluxes within the model are denoted as boxes and dashed lines; the diagram is simplified as compared to the underlying CLASSIC model for ease of visualization. *Note that forest product fluxes are not necessarily localized within Canada due to the export of forest products, but are localized within Canada in the CLASSIC model. **Note that CLASSIC does not yet explicitly represent the peatland carbon pool (~190 Pg; Table S4; Appendix S1; Results & Discussion).

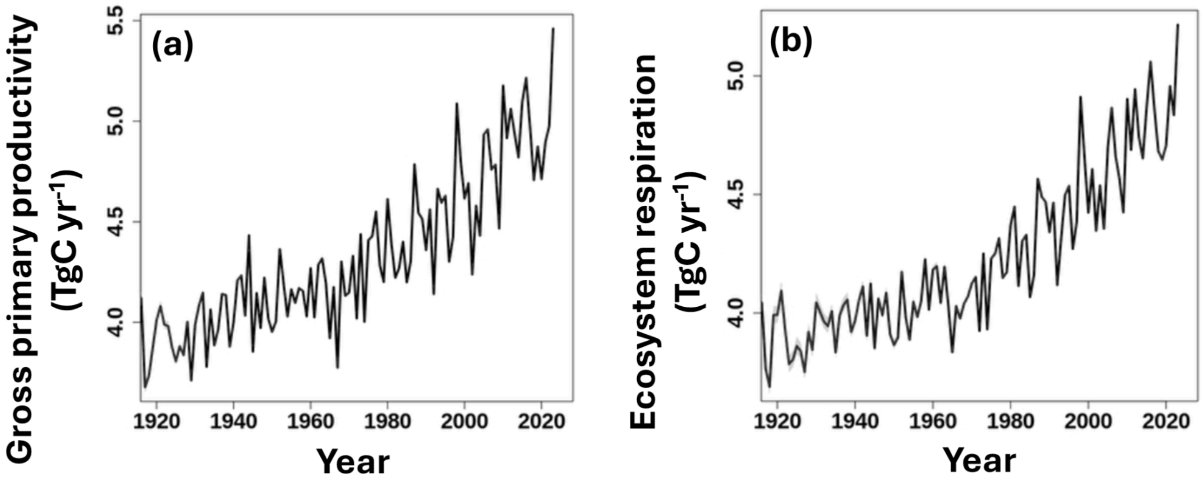

Figure S11: Plots of **a)** gross primary productivity and **b)** ecosystem respiration Canada-wide from four CLASSIC runs (See Table S3, run #1 - 4). The shaded regions show the minimum and maximum across runs (note there is limited variation in these quantities between runs).

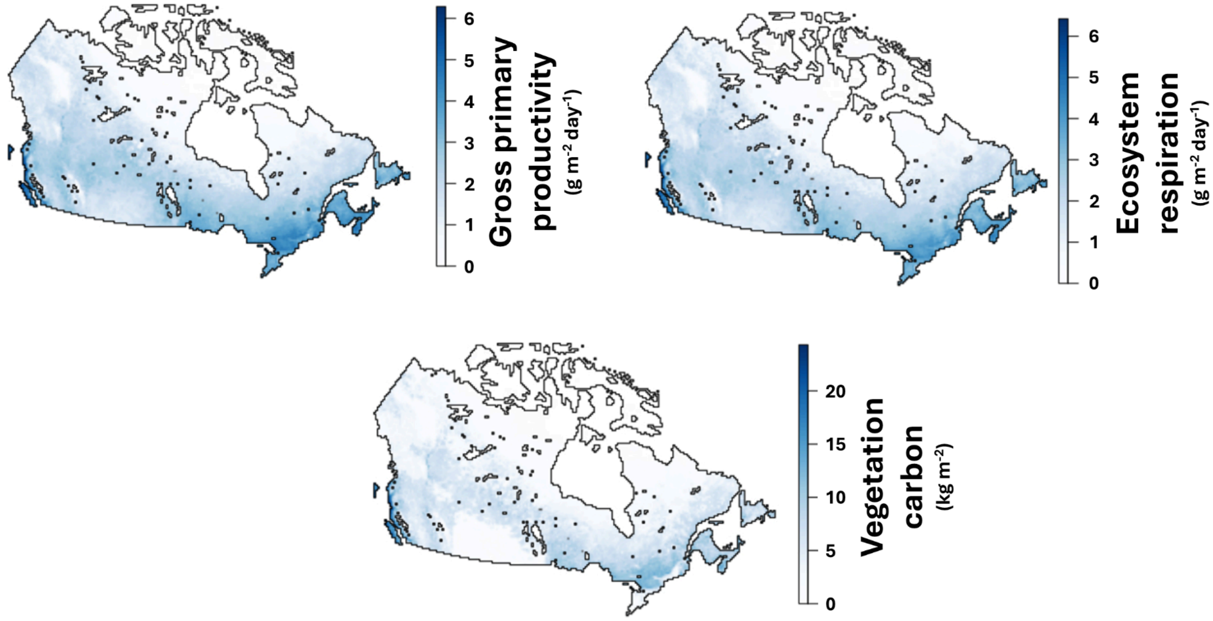

Figure S12: The spatial distribution of gross primary productivity, ecosystem respiration, and vegetation biomass Canada-wide averaged from 2002 to 2022 from four CLASSIC runs (See Table S3, run #1 - 4).

Tables:

Table S1: Overview of independent data sets.

Dataset	Variables[1]	Method	Period	Extent	Spatial resolution[2]	Temporal resolution[2]	References
FluxCom	GPP	machine learning ensemble	1980–2013	Canada wide	0.5 degree	daily	Jung et al., 2019
MODIS	GPP	light use efficiency model	2000–2016, 2000–2018	Canada wide	1 km	8 day	Zhang et al., 2017
GOSIF	GPP	statistical model	2000–2018	Canada wide	0.05 degree	8 day	Li et al., 2019
GLASS	GPP	light use efficiency model	1982-2019	Canada wide	0.05 degree	8 day	Liang et al., 2021
GEOCARBON	AGB	machine learning	Snapshot	Forests	1 km	snapshot	Avitabile et al., 2016; Santoro et al., 2015
Zhang	AGB	data fusion	Snapshot	Forests	1 km	snapshot	Zhang et al., 2020
FOSXue	AGB	upscaled insitu measurement	1999–2018	Canada wide	1 km		Schepaschenko et al., 2019; Xue et al., 2017
Huang2021	AGB	remote sensed SAR	Snapshot	Forests	1 km	snapshot	Huang et al., 2021
HWSD	CSOIL	soil inventory	Snapshot	Canada wide	1 km	snapshot	Todd-Brown et al., 2013
SG250m	CSOIL	machine learning	Snapshot	Canada wide	250 m	snapshot	Hengl et al., 2017
CAMS	NBP	atmospheric inversion	1979-2019	Canada wide	~2 degrees	3 hour	Agusti-Panareda et al., 2019
CarboScope	NBP	atmospheric inversion	1999-2019	Canada wide	2 degrees	daily	Rodenbeck et al., 2018
CT2019	NBP	atmospheric inversion	2000-2018	Canada wide	0.5 degree	monthly	Jacobson et al., 2020
Ctracker-LA	NBP	atmospheric inversion	2007-2015	Canada wide	10 km	3 hour	Hu et al., 2019
inTech	NBP	ecosystem model (aggregated and spatially explicit)	1901-2008	Forests	1 km	annual	Chen et al., 2000; 2003
RECCAP2	NBP	Bottom up models (n = 19), inversions (n = 7)	2000 - 2019	Canada wide	100 - 300 km	various	Poulter et al., 2025
GFED4.1	fFire	bottom up model using burned area	2003-2015	Canada wide	0.25 degree	monthly	Giglio et al., 2013
FINN2.5	fFire	bottom up model using active fire observations	2003-2015	Canada wide	0.1 degree	daily	Wiedinmyer et al., 2011
FEER1.0-G1.2	fFire	top down derived using smoke aerosol	2003-2015	Canada wide	0.1 degree	daily	Ichoku et al., 2014
QFED2.4r1	fFire	top down derived using smoke aerosol	2003-2015	Canada wide	0.1 degree	daily	Koster et al., 2015
CT2019	fFire	bottom up model using burned area	2003-2015	Canada wide	0.5 degree	monthly	Van der Werf et al., 2017; 2010

[1]These acronyms are defined in section 3.3 and Appendix S1.

[2]Several data sets below are available in multiple spatial and temporal resolutions. The table provides the resolution used herein; in some cases, other combinations of spatial and temporal resolutions are available.

Table S2: Detailed setup and other run information for FLUXNET sites included in the site-level comparison suite.

Site code	Lat,Lon (dd.dd)	Flux obs. years	Biomass obs. years	Simulated disturbance	Disturbance narrative details	Simulated PFT cover[2]	PFT cover narrative details	References
CA-Ca1	49.9,-125.3	1996 - 2010	2002	1940 (harvest)	50% harvest in 1937 and remaining 50% harvested in 1943.	100% NdlEvgTr	80% douglas-fir, 17% western red cedar, 3% western hemlock, sparse understory.	Humphreys et al., 2006; Pastorello et al., 2020
CA-Ca2	49.9,-125.3	1999 - 2010	2002	1940 (harvest), 2000 (harvest)	-	100% NdlEvgTr	93% douglas-fir, 7% western red cedar, dense understory.	Humphreys et al., 2006; Pastorello et al., 2020
CA-Ca3	49.5,-124.9	2001 - 2010	2002	1937 (harvest), 1938 (burn), 1987 (harvest), 1988 (burn)	100% harvest and slash burned in 1937 and 1987.	100% NdlEvgTr	75% douglas-fir, 21% western red cedar, 4% grand fir, dense understory, differences in composition before fire.	Humphreys et al., 2006; Pastorello et al., 2020
CA-Cbo	44.3,-79.9	1994 - 2014	-	1896 (harvest)	agricultural abandonment in 1896 as inferred from stand age (100 years old in 1996).	17% NdlEvgTr, 83% BdlDCoTr	52.2% red maple, 13.5 % is eastern white pine, 7% is large-tooth aspen, 7% is white ash, 20% other hardwood.	Teklemariam et al., 2009
CA-Gro	48.2,-82.2	2003 - 2014	2003	1930 (harvest)	-	6.49% NdlEvgTr, 1% NdlDcdTr, 17.62% CNEvgTr, 58.88% BdlDCoTr	Dominated by trembling aspen, black spruce, white spruce, white birch, and balsam fir.	McCaughey et al., 2006; Pastorello et al., 2020
CA-Man	55.9,-98.5	1994 - 2008	1994	1839 (burn)	100% burned in 1839 as inferred from stand age (i.e. 155 years old in 1994).	90% CNEvgTr, 5% BdlEvgSh, 5% BdlDCoSh	Black spruce with understory of feather moss and labrador tea.	Bergeron et a., 2007; Pastorello et al., 2020
CA-NS1	55.9,-98.5	2001 - 2005	2003	1850 (burn)	-	100% CNEvgTr	Black spruce.	Goulden et al., 2006; Goulden et al., 2011
CA-NS2	55.9,-98.5	2001 - 2005	2003	1930 (burn)	-	100% CNEvgTr	Black spruce.	Goulden et al., 2006; Goulden et al., 2011
CA-NS3	55.9,-98.4	2001 - 2005	2003	1964 (burn)	-	100% CNEvgTr	Post fire mix of herbs, shrubs, aspen, black spruce, and jack pine which is expected to return to black spruce.	Goulden et al., 2006; Goulden et al., 2011
CA-NS4	55.9,-98.4	2002 - 2005	-	1964 (burn)	-	100% CNEvgTr	Post fire mix of herbs, shrubs, aspen, black spruce, and jack pine which is expected to return to black spruce.	Goulden et al., 2006; Goulden et al., 2011
CA-NS5	55.9,-98.5	2001 - 2005	2003	1981 (burn)	-	100% CNEvgTr	Post fire mix of herbs, shrubs, aspen, black spruce, and jack pine which is expected to return to black spruce.	Goulden et al., 2006; Goulden et al., 2011
CA-NS6	55.9,-99	2001 - 2005	2003	1989 (burn)	-	100% CNEvgTr	Post fire mix of herbs, shrubs, aspen, black spruce, and jack pine which is expected to return to black spruce.	Goulden et al., 2006; Goulden et al., 2011
CA-NS7	56.6,-99.9	2002 - 2005	2003	1998 (burn)	-	100% CNEvgTr	Post fire mix of herbs, shrubs, aspen, black spruce, and jack pine which is expected to return to black spruce.	Goulden et al., 2006; Goulden et al., 2011
CA-Oas	53.6,-106.2	1996 - 2010	2004	1919 (burn)	100% of footprint burned in 1919. Years after tent caterpillar defoliation in 2016 not included.	100% BdlDCoTr	90% trembling aspen, 10% balsam poplar, hazelnut understory (50% of LAI).	Stephens et al., 2018; Pastorello et al., 2020
CA-Obs	54,-105.1	1997 - 2010	-	1894 (burn)	-	90% CNEvgTr, 10% NdlDcdTr	90% black spruce, 10% tamarack.	Bergeron et al., 2007; Pastorello et al., 2020
CA-Qc2	49.8,-74.6	2007 - 2010	-	1975 (harvest), 1998 (harvest 10%)	100% harvested in 1975 and pre-commercial thinning of ~15% of trees in 70% of the footprint in 1998.	90% CNEvgTr, 10% BdlEvgSh	90% black spruce, tamarack in humid areas.	Payeur-Poirier et al., 2012
CA-Qfo	49.7,-74.3	2003 - 2010	2004	1905 (burn)	-	90% CNEvgTr, 10% BdlDCoSh	Black spruce, with a few jack pine, tamarack, and alder in wet areas.	Bergeron et al., 2007; Pastorello et al., 2020
CA-SF1	54.5,-105.8	2003 - 2006	2005	1977 (burn)	-	100% NdlEvgTr	Jack pine, black spruce, and trembling aspen.	Mkhabela et al., 2009; Pastorello et al., 2020

Table S2 contd.

Site code	Lat,Lon (dd.dd)	Flux obs. years	Biomass obs. years	Simulated disturbance	Disturbance narrative details	Simulated PFT cover[2]	PFT cover narrative details	References
CA-SF2	54.3,-105.9	2001 - 2006	2001,	1989 (burn)	-	100% NdlEvgTr	Jack pine, black spruce, and trembling aspen.	Mkhabela et al., 2009; Pastorello et al.,
CA-SF3	54.1,-106	2001 - 2006	2001, 2005	1998 (burn)	-	100% NdlEvgTr	Jack pine, black spruce, and trembling aspen.	Mkhabela et al., 2009; Pastorello et al., 2020
CA-SJ2	53.9,-104.6	2002 - 2010	2002	2002 (harvest)	-	100% NdlEvgTr	Jack pine.	Mkhabela et al., 2009; Pastorello et al., 2020
CA-TP1	42.7,-80.6	2002 - 2017	2004, 2007[1]	2002 (harvest)	Planted from bare ground in 2002.	98% NdlEvgTr, 2% BdlDCoTr	Eastern white pine.	Peichl et al., 2010; Pastorello et al., 2020
CA-TP2	42.8,-80.5	2002 - 2008	2004, 2007[1]	1989 (harvest)	Planted from bare ground in 1989.	98% NdlEvgTr, 2% BdlDCoTr	Eastern white pine.	Peichl et al., 2010; Pastorello et al., 2020
CA-TP3	42.7,-80.3	2002 - 2017	2004, 2007[1]	1974 (harvest)	Planted from bare ground in 1974.	99% NdlEvgTr, 1% BdlDCoTr	92% eastern white pine, 5% jack pine, 1% oak	Peichl et al., 2006; Pastorello et al., 2020
CA-TP4	42.7,-80.4	2002 - 2017	2004, 2007[1]	1939 (harvest), 1983 (harvest 21%)	Planted from bare ground in 1939 and ~25% of trees in 84% of the footprint thinned in 1983.	93% NdlEvgTr, 7% BdlDCoTr	82% eastern white pine, 11% balsam fir, 4% oak, understory of 2% red maple and 2% wild black cherry.	Peichl et al., 2006; Pastorello et al., 2020
CA-TPD	42.6,-80.6	2012 - 2014	-	1929 (harvest)	Agricultural abandonment in 1929 as inferred from stand age (i.e. 90 years old in 2019).	5% NdlEvgTr, 95% BdlDCoTr	White oak, scattered sugar, red maple, american beech, black oak, red oak, white ash, white and red pine.	Beamesderfer et a., 2020

[1]Surveys from 2005 and 2006 don't include understory biomass and are therefore excluded.

[2]PFT codes: needleleaf evergreen tree (NdlEvgTr), needleleaf deciduous tree (NdlDcdTr), continental needleleaf evergreen tree (CNEvgTr), broadleaf cold deciduous tree (BdlDCoTr), broadleaf evergreen shrubs (BdlEvgSh), broadleaf deciduous cold shrubs (BdlDCoSh). Also see Curasi et al.[1].

Table S3: Overview of CLASSIC model runs.

#	Type	Years	ISIMIP climate forcing[1]	Atmospheric CO_2[2]	Disturbance[3]	Figures[4]
1	Historical	1740 - 2023	Reanalysis (CRU-JRA)	global carbon project	NFIS with disturbance infered from 1920's stand age prior to 1918	3-6, S3, S6, S9-S12
2	Historical	1740 - 2023	Reanalysis (CRU-JRA)	global carbon project	NFIS with 1920 - 1930 average disturbance prior to 1918	3-6, S3, S6, S9-S12
3	Historical	1740 - 2023	Reanalysis (CRU-JRA)	global carbon project	Vector data with disturbance infered from 1920's stand age prior to 1918	3-6, S3, S6, S9-S12
4	Historical	1740 - 2023	Reanalysis (CRU-JRA)	global carbon project	Vector data with 1920 - 1930 average disturbance prior to 1918	3-6, S3, S6, S9-S12
5	Factorial	1740 - 2023	Reanalysis (CRU-JRA)	Held constant	None	4
6	Factorial	1740 - 2023	Reanalysis (CRU-JRA)	global carbon project	None	4
7	Factorial	1740 - 2023	Reanalysis (CRU-JRA)	global carbon project	NFIS with disturbance infered from 1920's stand age prior to 1918, none after 1940	4
8	Factorial	1740 - 2023	Reanalysis (CRU-JRA)	global carbon project	NFIS with 1920 - 1930 average disturbance prior to 1918, none after 1940	4
9	Factorial	1740 - 2023	Reanalysis (CRU-JRA)	global carbon project	Vector data with disturbance infered from 1920's stand age prior to 1918, none after 1940	4
10	Factorial	1740 - 2023	Reanalysis (CRU-JRA)	global carbon project	Vector data with 1920 - 1930 average disturbance prior to 1918, none after 1940	4
11	Factorial	1740 - 2023	Reanalysis (CRU-JRA)	global carbon project	NFIS with disturbance infered from 1920's stand age prior to 1918, none after 2002	4
12	Factorial	1740 - 2023	Reanalysis (CRU-JRA)	global carbon project	NFIS with 1920 - 1930 average disturbance prior to 1918, none after 2002	4
13	Factorial	1740 - 2023	Reanalysis (CRU-JRA)	global carbon project	Vector data with disturbance infered from 1920's stand age prior to 1918, none after 2002	4
14	Factorial	1740 - 2023	Reanalysis (CRU-JRA)	global carbon project	Vector data with 1920 - 1930 average disturbance prior to 1918, none after 2002	4
15	Factorial	1740 - 2023	Reanalysis (CRU-JRA)	global carbon project	NFIS, none prior to 1940	4, S9
16	Factorial	1740 - 2023	Reanalysis (CRU-JRA)	global carbon project	Vector, none prior to 1940	4, S9
17	Factorial	1740 - 2023	Reanalysis (CRU-JRA)	global carbon project	NFIS, none prior to 2002	4
18	Factorial	1740 - 2023	Reanalysis (CRU-JRA)	global carbon project	Vector, none prior to 2002	4

[1]For details of the CRU-JRA forcing, see Friedlingstein et al.[9] and Wang et al.,[10].

[2]For details of the global carbon project, atmospheric CO_2 concentrations see Friedlingstein et al.[11].

[3]For details of the disturbance drivers, see Beaver et al.[6].

[4]Denotes the model runs associated with particular figures

Table S4: Comparisons between wall-to-wall carbon pool estimates from CLASSIC and those from Sothe et al., 2022[2], and carbon2018[12]. Carbon2018 includes estimates for forests only.

Pool	Sothe et al., 2022	carbon2018	CLASSIC	2022 (forest only)	carbon2018 (forest only)	CLASSIC (forest only)
Litter	2.60	-	22.3	-	16.3	14.5
Soil (including peat)	572[1]	262[2]	-	-	-	-
Soil (excluding peat)	382[1]	120[2]	208	-	-	146
Belowground biomass	4.30	-	7.5	4.30	2.75	5.9
Aboveground biomass	14.2	-	21.4	14.0	11.2	17.9
Vegetation carbon	18.5	-	28.9	18.3	13.9	23.8

[1]Soil carbon estimates extend to a depth of 2m
[2]Soil carbon estimates extend to a depth of 1m

References:

1. Curasi, S. R. *et al.* Evaluating the performance of the Canadian Land Surface Scheme Including Biogeochemical Cycles (CLASSIC) tailored to the pan-Canadian domain. *Journal of Advances in Modeling Earth Systems* **15**, e2022MS003480 (2023).
2. Sothe, C. *et al.* Large soil carbon storage in terrestrial ecosystems of Canada. *Global Biogeochem. Cycles* **36**, (2022).
3. Arora, V., Seiler, C., Wang, L. & Kou‐Giesbrecht, S. Towards an ensemble-based evaluation of land surface models in light of uncertain forcings and observations. *Biogeosciences* (2023) doi:10.5194/bg-20-1313-2023.
4. Jones, C. D. *et al.* C4MIP–The coupled climate–carbon cycle model intercomparison project: Experimental protocol for CMIP6. *Geoscientific Model Development* **9**, 2853–2880 (2016).
5. Arora, V. K. *et al.* Carbon–concentration and carbon–climate feedbacks in CMIP5 Earth system models. *J. Clim.* **26**, 5289–5314 (2013).

6. Beaver, J. *et al.* High-resolution Canada domain disturbance forcings suitable for land surface modeling applications. *Scientific Data* (2025) doi:10.21203/rs.3.rs-6025328/v1.

7. Curasi, S. R., Melton, J. R., Arora, V. K., Humphreys, E. R. & Whaley, C. H. Global climate change below 2 °C avoids large end century increases in burned area in Canada. *Npj Clim. Atmos. Sci.* **7**, 1–11 (2024).

8. Chen, J. M. *et al.* Spatial distribution of carbon sources and sinks in Canada's forests. *Tellus B Chem. Phys. Meteorol.* **55**, 622–641 (2003).

9. Friedlingstein, P. *et al.* Global carbon budget 2022. *Earth System Science Data* **14**, 4811–4900 (2022).

10. Wang, L., Arora, V. K., Bartlett, P., Chan, E. & Curasi, S. R. Mapping of ESA-CCI land cover data to plant functional types for use in the CLASSIC land model. *Biogeosciences* **20**, 2265–2282 (2023).

11. Friedlingstein, P. *et al.* Global carbon budget 2021. *Earth System Science Data* **14**, 1917–2005 (2022).

12. Lajtha, K., Bailey, V. L. & McFarlane, K. The Second State of the Carbon Cycle Report-Chapter 12. Soils. (2018).

13. van der Werf, G. R. *et al.* Global fire emissions and the contribution of deforestation, savanna, forest, agricultural, and peat fires (1997–2009). *Atmos. Chem. Phys. Discuss.* **10**, 16153–16230 (2010).

14. van der Werf, G. R. *et al.* Global fire emissions estimates during 1997–2016. *Earth Syst. Sci. Data* **9**, 697–720 (2017).

15. Koster, R. D., Darmenov, A. S. & da Silva, A. M. *The Quick Fire Emissions Dataset (QFED): Documentation of Versions 2.1, 2.2 and 2.4*. https://ntrs.nasa.gov/citations/20180005253 (2015).

16. Ichoku, C. & Ellison, L. Global top-down smoke-aerosol emissions estimation using satellite fire radiative power measurements. *Atmos. Chem. Phys.* **14**, 6643–6667 (2014).

17. Wiedinmyer, C. *et al.* The Fire INventory from NCAR (FINN): a high resolution global model to estimate the emissions from open burning. *Geosci. Model Dev.* **4**, 625–641 (2011).

18. Giglio, L., Randerson, J. T. & van der Werf, G. R. Analysis of daily, monthly, and annual burned area using the fourth-generation global fire emissions database (GFED4). *J. Geophys. Res. Biogeosci.* **118**, 317–328 (2013).

19. Chen, W., Chen, J., Liu, J. & Cihlar, J. Approaches for reducing uncertainties in regional forest carbon balance. *Global Biogeochem. Cycles* **14**, 827–838 (2000).

20. Rödenbeck, C., Zaehle, S., Keeling, R. & Heimann, M. How does the terrestrial carbon exchange respond to inter-annual climatic variations? A quantification based on atmospheric CO 2 data. *Biogeosciences* **15**, 2481–2498 (2018).

21. Agustí-Panareda, A. *et al.* Modelling CO 2 weather–why horizontal resolution matters. *Atmospheric Chemistry and Physics* **19**, 7347–7376 (2019).

22. Hengl, T. *et al.* SoilGrids250m: Global gridded soil information based on machine learning. *PLoS One* **12**, e0169748 (2017).

23. Todd-Brown, K. E. O. *et al.* Causes of variation in soil carbon simulations from CMIP5 Earth system models and comparison with observations. *Biogeosciences* **10**, 1717–1736 (2013).

24. Huang, Y. *et al.* A global map of root biomass across the world's forests. *Earth Syst. Sci. Data* **13**, 4263–4274 (2021).

25. Xue, B.-L. *et al.* Evaluation of modeled global vegetation carbon dynamics: Analysis based on global carbon flux and above-ground biomass data. *Ecol. Modell.* **355**, 84–96 (2017).

26. Schepaschenko, D. *et al.* The Forest Observation System, building a global reference dataset for remote sensing of forest biomass. *Sci Data* **6**, 198 (2019).

27. Zhang, Y. & Liang, S. Fusion of Multiple Gridded Biomass Datasets for Generating a Global Forest Aboveground Biomass Map. *Remote Sensing* **12**, 2559 (2020).

28. Santoro, M. *et al.* Forest growing stock volume of the northern hemisphere: Spatially explicit estimates for 2010 derived from Envisat ASAR. *Remote Sens. Environ.* **168**, 316–334 (2015).

29. Avitabile, V. *et al.* An integrated pan-tropical biomass map using multiple reference datasets. *Glob. Chang. Biol.* **22**, 1406–1420 (2016).

30. Liang, S. *et al.* The Global Land Surface Satellite (GLASS) Product Suite. *Bull. Am. Meteorol. Soc.* **102**, E323–E337 (2021).

31. Li, X. & Xiao, J. A Global, 0.05-Degree Product of Solar-Induced Chlorophyll Fluorescence Derived from OCO-2, MODIS, and Reanalysis Data. *Remote Sensing* **11**, 517 (2019).

32. Zhang, Y. *et al.* A global moderate resolution dataset of gross primary production of vegetation for 2000–2016. *Scientific Data* **4**, 1–13 (2017).

33. Jung, M. *et al.* The FLUXCOM ensemble of global land-atmosphere energy fluxes. *Sci Data* **6**, 74 (2019).

34. Humphreys, E. R. *et al.* Carbon dioxide fluxes in coastal Douglas-fir stands at different stages of development after clearcut harvesting. *Agric. For. Meteorol.* **140**, 6–22 (2006).

35. Teklemariam, T., Staebler, R. M. & Barr, A. G. Eight years of carbon dioxide exchange above a mixed forest at Borden, Ontario. *Agric. For. Meteorol.* **149**, 2040–2053 (2009).

36. McCaughey, J. H., Pejam, M. R., Arain, M. A. & Cameron, D. A. Carbon dioxide and energy fluxes from a boreal mixedwood forest ecosystem in Ontario, Canada. *Agric. For. Meteorol.* **140**, 79–96 (2006).

37. Mathys, A. *et al.* Carbon balance of a partially harvested mixed conifer forest following mountain pine beetle attack and its comparison to a clear-cut. *Biogeosciences* **10**, 5451–5463 (2013).

38. Bergeron, O. *et al.* Comparison of carbon dioxide fluxes over three boreal black spruce forests in Canada. *Glob. Chang. Biol.* **13**, 89–107 (2007).

39. Goulden, M. L. *et al.* An eddy covariance mesonet to measure the effect of forest age on land-atmosphere exchange. *Glob. Chang. Biol.* **12**, 2146–2162 (2006).

40. Goulden, M. L. *et al.* Patterns of NPP, GPP, respiration, and NEP during boreal forest succession: CARBON DYNAMICS DURING BOREAL SUCCESSION. *Glob. Chang. Biol.* **17**, 855–871 (2011).

41. Stephens, J. J. *et al.* Effects of forest tent caterpillar defoliation on carbon and water fluxes in a boreal aspen stand. *Agric. For. Meteorol.* **253-254**, 176–189 (2018).

42. Mkhabela, M. S. *et al.* Comparison of carbon dynamics and water use efficiency following fire and harvesting in Canadian boreal forests. *Agric. For. Meteorol.* **149**, 783–794 (2009).

43. Pastorello, G. *et al.* The FLUXNET2015 dataset and the ONEFlux processing pipeline for eddy covariance data. *Sci. Data* **7**, (2020).

44. Peichl, M., Arain, M. A. & Brodeur, J. J. Age effects on carbon fluxes in temperate pine forests. *Agric. For. Meteorol.* **150**, 1090–1101 (2010).

45. Peichl, M. & Arain, M. A. Above- and belowground ecosystem biomass and carbon pools in an age-sequence of temperate pine plantation forests. *Agric. For. Meteorol.* **140**, 51–63 (2006).

46. Beamesderfer, E. R., Arain, M. A., Khomik, M., Brodeur, J. J. & Burns, B. M. Response of carbon and water fluxes to meteorological and phenological variability in two eastern North American forests of similar age but contrasting species composition–a multiyear comparison. *Biogeosciences* **17**, 3563–3587 (2020).

47. Payeur-Poirier, J.-L., Coursolle, C., Margolis, H. A. & Giasson, M.-A. CO2 fluxes of a boreal black spruce chronosequence in eastern North America. *Agric. For. Meteorol.* **153**, 94–105 (2012).

48. Jacobson, A. R. *et al.* CarbonTracker documentation CT2019 release. *Global Monitoring Laboratory-Carbon Cycle Greenhouse Gases* (2020).

49. Hu, L. *et al.* Enhanced North American carbon uptake associated with El Niño. *Sci. Adv.* **5**, eaaw0076 (2019).

50. Poulter, B. *et al.* The North American greenhouse gas budget: Emissions, removals, and integration for CO_2, CH_4, and N_2O (2010–2019): Results from the Second REgional Carbon Cycle Assessment and processes study (RECCAP2). *Global Biogeochem. Cycles* **39**, (2025).
